# Mystic Acetaldehyde: The Never-Ending Story on Alcoholism

**DOI:** 10.3389/fnbeh.2017.00081

**Published:** 2017-05-11

**Authors:** Alessandra T. Peana, María J. Sánchez-Catalán, Lucia Hipólito, Michela Rosas, Simona Porru, Federico Bennardini, Patrizia Romualdi, Francesca F. Caputi, Sanzio Candeletti, Ana Polache, Luis Granero, Elio Acquas

**Affiliations:** ^1^Department of Chemistry and Pharmacy, University of SassariSassari, Italy; ^2^Department of Pharmacy, Pharmaceutical Technology and Parasitology, University of ValenciaValència, Spain; ^3^Department of Life and Environmental Sciences, University of CagliariCagliari, Italy; ^4^Department of Pharmacy and Biotechnology, University of BolognaBologna, Italy; ^5^Centre of Excellence on Neurobiology of Addiction, University of CagliariCagliari, Italy

**Keywords:** ethanol, acetaldehyde, salsolinol, ethanol metabolism, epigenetics, neuroinflammation, mesolimbic system, dopamine

## Abstract

After decades of uncertainties and drawbacks, the study on the role and significance of acetaldehyde in the effects of ethanol seemed to have found its main paths. Accordingly, the effects of acetaldehyde, after its systemic or central administration and as obtained following ethanol metabolism, looked as they were extensively characterized. However, almost 5 years after this research appeared at its highest momentum, the investigations on this topic have been revitalized on at least three main directions: (1) the role and the behavioral significance of acetaldehyde in different phases of ethanol self-administration and in voluntary ethanol consumption; (2) the distinction, in the central effects of ethanol, between those arising from its non-metabolized fraction and those attributable to ethanol-derived acetaldehyde; and (3) the role of the acetaldehyde-dopamine condensation product, salsolinol. The present review article aims at presenting and discussing prospectively the most recent data accumulated following these three research pathways on this never-ending story in order to offer the most up-to-date synoptic critical view on such still unresolved and exciting topic.

## Introduction

The investigations on the role of acetaldehyde and ethanol metabolism in the central effects of ethanol have been a long-standing issue of interest and controversy (McBride et al., 2002; Quertemont et al., 2005; Correa et al., 2012). Thus, although numerous lines of research have focused on the role of acetaldehyde in different aspects of ethanol effects, the role of its main metabolite in the biological basis of its effects and, in particular, of its reinforcing properties, are still not fully understood. Accordingly, contrasting theories have arisen suggesting, on one hand, that ethanol is a molecule responsible of the reinforcing properties of alcoholic drinks and, on the other hand, that ethanol acts as pro-drug and hence owns most of its central effects to other compounds generated, directly or indirectly, from its metabolism. Followers of the first view suggest that ethanol exerts its properties within the brain by affecting numerous neurotransmitter systems, that there is no significant evidence that its metabolites cross the blood brain barrier and that the metabolites occur for only short periods to mediate the effects of ethanol intoxication. The working hypothesis that envisions ethanol as a pro-drug, on the other hand, suggests that its activating and reinforcing properties are supported by the central actions of its metabolites (Deng and Deitrich, 2008; Deitrich, 2011; Karahanian et al., 2011; Correa et al., 2012; Hipólito et al., 2012; Peana and Acquas, 2013; Israel et al., 2015), no matter if generated centrally or peripherally. In the latter case, according to the pro-drug theory, acetaldehyde’s plasma levels, following ethanol intake, would reach concentrations sufficient to significantly affect its targets within the central nervous system (CNS). Another possibility predicts that the activating and reinforcing properties, mostly mediated through the involvement of the dopaminergic mesolimbic system, could depend on the actions of ethanol itself in combination with those of its metabolites produced within the brain from local metabolism of ethanol (Sánchez-Catalán et al., 2008; Hipólito et al., 2009; Martí-Prats et al., 2013, 2015). Accordingly, after ethanol administration, the net effect on the activity of dopamine (DA) neurons would be the *algebraic* consequence of the activation, due to the action of the ethanol derivatives, and the depression, due to ethanol itself (Martí-Prats et al., 2013, 2015). Indeed, the concentrations and duration of the effects of ethanol and its derivatives determine the final effect on DA neurons, which is ultimately governed by the rate of ethanol metabolism (Martí-Prats et al., 2013, 2015).

Significant behavioral evidence implicates acetaldehyde in the mechanisms underlying the psychopharmacological effects of ethanol (Correa et al., 2012; Peana and Acquas, 2013; Peana et al., 2016). Acetaldehyde has reinforcing properties on its own (Correa et al., 2012; Peana et al., 2016), induces euphoria at low concentrations (Eriksson, 2001) and has been involved in alcohol addiction (Deng and Deitrich, 2008; Deehan et al., 2013). Moreover, support to the critical role of acetaldehyde in the reinforcing properties of ethanol was provided by the observations that a negative interference with the peripheral or central metabolism of ethanol to acetaldehyde, as well as a reduction of its bioavailability, prevents several ethanol actions, including its reinforcing effects (Foddai et al., 2004; Melis et al., 2007; Peana et al., 2008, 2010a,b, 2015; Enrico et al., 2009; Martí-Prats et al., 2013, 2015; Orrico et al., 2013, 2014). This is in agreement with the original observation, made by Chevens (1953). In fact, he reported that his patients did not perceive aversive effects by taking low amounts of ethanol when they were under treatment with disulfiram, an inhibitor of aldehyde dehydrogenase (ALDH), suggesting that ALDH inhibition could increase the euphoric and pleasurable effects of small doses of ethanol by increasing acetaldehyde’s availability (Brown et al., 1980).

In addition to the above, acetaldehyde has multiple tissue damage effects and these also should be appreciated as a feature of another never-ending story. In fact, humans are frequently exposed to acetaldehyde from various sources including alcoholic beverages, tobacco smoke and foods and even microbes are responsible for the bulk of acetaldehyde production from ethanol both in saliva and in the Helicobacter pylori-infected and achlorhydric stomach (Salaspuro, 2011). Moreover, acetaldehyde is also usually used as a food additive and aroma agent. Unfortunately, acetaldehyde is mutagenic and carcinogenic being responsible of DNA damage and of several cancer-promoting effects (Dellarco, 1988; Seitz and Stickel, 2010). Accordingly, acetaldehyde and ethanol are two of the compounds for which the most comprehensive evidence on epidemiology and mechanisms of carcinogenesis is accessible. In the relationship between alcohol consumption and development of different forms of cancer, the impact of the risk of developing this pathology mostly depends on alcohol consumption (Shield et al., 2013) and even a moderate drinking has been shown to cause cancer (Bagnardi et al., 2013). Different hypothesis have been proposed to explain how ethanol and acetaldehyde may cause or contribute to carcinogenesis, the main mechanism being attributable to the metabolism of ethanol into the carcinogenic, and DNA binding, acetaldehyde (Seitz and Stickel, 2007). Accordingly, humans deficient in mitochondrial ALDH2 present an increased risk of developing malignant tumours of the upper digestive tract (Lachenmeier and Salaspuro, 2017). Likewise, ethanol may also be metabolized into acetaldehyde by cytochrome CYP2E1, a process that produces radical oxygen species (ROS) that may lead to lipid peroxidation and to the formation of mutagenic adducts (Pflaum et al., 2016). Additionally, acetaldehyde may also lead to DNA hypomethylation, which changes the expression of oncogenes and tumour-suppression genes (Seitz and Stickel, 2007; Pflaum et al., 2016). Finally, in this regard, recent research from Lachenmeier and Salaspuro (2017) reported that many of previous animal toxicology-based risk assessments might have underestimated the risk of acetaldehyde toxicity. Interestingly, buccal tablets slowly releasing L-cysteine, a semi-essential amino acid, are able to reduce or remove microbially-formed carcinogenic acetaldehyde from saliva during ethanol intake. Indeed, L-cysteine binds covalently acetaldehyde producing a stable compound (Salaspuro et al., 2002).

Another critical issue related to the neurobiological basis of the central effects of ethanol refers to the increasing evidence of other biologically active compounds (adducts), which are obtained after acetaldehyde’s reaction with endogenous monoamines and appear responsible of ethanol’s effects. As regards these adducts, the properties of salsolinol (formed when acetaldehyde binds to DA) as well as its potential role in the neurobiological properties of ethanol have been recently re-evaluated not only in light of the reinforcing properties of ethanol (Hipólito et al., 2012; Deehan et al., 2013) but also in light of the ability of salsolinol itself to affect ethanol intake (Quintanilla et al., 2014), locomotor activity (Hipólito et al., 2010; Quintanilla et al., 2014), conditioned place preference (CPP; Matsuzawa et al., 2000; Hipólito et al., 2011) and to exert neurotoxicity (Hernández et al., 2016).

In this regard, while many studies link Parkinson’s disease with exposure to endogenous salsolinol (Tieu, 2011), this molecule has recently also been suggested as responsible for inducing experimental enteric neurodegeneration in rats (Kurnik et al., 2015).

## The Issue of Acetaldehyde Determination

During the last years, several studies attempted to measure acetaldehyde in blood and brain following the systemic administration of either ethanol or acetaldehyde itself in order to correlate plasma to brain concentrations. In particular, while some studies have described detection of brain acetaldehyde after peripheral ethanol (Kiessling, 1962; Sippel, 1974; Tabakoff et al., 1976; Eriksson and Sippel, 1977; Westcott et al., 1980; Hamby-Mason et al., 1997; Peana et al., 2008, 2010b) or acetaldehyde administration (Heap et al., 1995; Ward et al., 1997; Quertemont et al., 2004; Plescia et al., 2015), others reported failure to detect it after administration of either ethanol (Sippel, 1974; Eriksson and Sippel, 1977; Jamal et al., 2007) or acetaldehyde itself (Peana et al., 2010a). Indeed, increases of acetate but not acetaldehyde were detected in human plasma after ethanol intake (Puig and Fox, 1984; Sarkola et al., 2002). These controversial results could be associated with a number of critical confounding factors that overall still limit the reliable detection of this compound. In fact, acetaldehyde formation in the brain is still subject to speculation due to the lack of a specific method able to accurately and directly assay its levels. One of these limitations is certainly represented by the fact that ALDH is more abundantly expressed with respect to the catalase-H_2_O_2_ (Zimatkin et al., 1992). Indeed, the most efficient isoform of this dehydrogenase, ALDH2, rapidly metabolizes acetaldehyde to acetate (Deitrich, 2004; Deng and Deitrich, 2008). Moreover, considering the factors that may interfere with acetaldehyde’s assessments in the brain, another aspect that should be taken into account is that acetaldehyde possesses a short elimination half-life and is a highly reactive electrophilic chemical that, thereby, is able to bind to nucleophilic structures to give condensation products. Lastly, acetaldehyde, like other volatile compounds, can easily cross the alveolar-capillary membrane of the lungs and be eliminated by exhalation (Eriksson and Sippel, 1977; Tardif, 2007) making it plausible that also this factor may contribute significantly to the difficulty of its detection.

Overall, this evidence indicates that after systemic administration of ethanol or acetaldehyde itself, acetaldehyde concentration must increase above a certain threshold level (i.e., above the detection limit of the available analytical approaches) in order to be reliably detected in the brain.

## Peripheral Generation of Acetaldehyde

**Figure 1:**
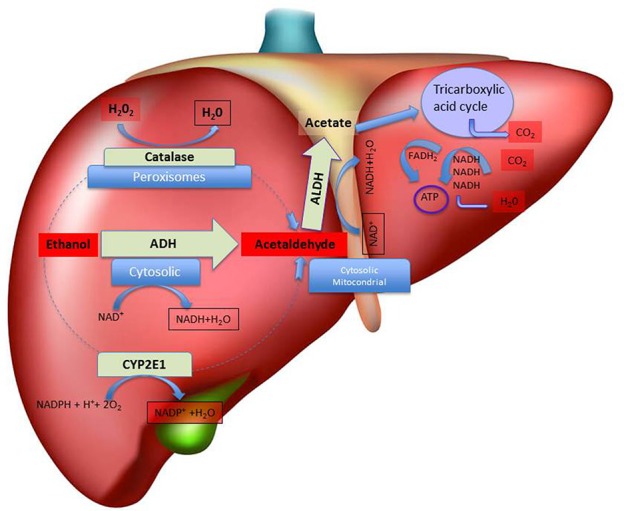
**Schematic representation of hepatic metabolism of ethanol.** The figure depicts, in the liver, the sub-cellular (cytosolic, peroxisomal and mitochondrial) localization of the main pathways of ethanol oxidative metabolism to acetaldehyde and of the main pathways of ethanol by-products (acetaldehyde and acetate) disposal, with indication of the relative co-factors involved. Abbreviations: ADH, Alcohol dehydrogenase; ALDH, Aldehyde dehydrogenase; ATP, adenosine triphosphate; CYP2E1, isoform 2E1 of cytochrome P_450_; FADH_2_, flavin-adenine dinucleotide coenzyme in its reduced form; NAD^+^, nicotinamide adenine dinucleotide coenzyme; NADPH, Nicotinamide Adenine Dinucleotide Phosphate coenzyme in its reduced form.

The conventional view that ethanol metabolism to acetaldehyde is carried out by class I liver alcohol dehydrogenase (ADH1; Haseba and Ohno, 2010), was obtained following animal (Bradford et al., 1993a,b; Escarabajal and Aragon, 2002; Peana et al., 2008) and human (Blomstrand and Theorell, 1970; Crow and Hardman, 1989; Sarkola et al., 2002) experiments with specific inhibitors (pyrazoles) of ADH (Figure 1). In humans, ADH1 is further classified into three subcategories, ADH1A, 1B and 1C (all inhibited by 4-methylpyrazole, 4-MP), which are the main ADHs for the oxidation of ethanol (Hempel et al., 1984). Interestingly, also class III (ADH3) has been reported to contribute to systemic ethanol metabolism in a dose-dependent manner, thereby contributing to diminish the consequences of acute ethanol intoxication. In particular, ADH3 (the isoform belonging to class III according to the old nomenclature may participate to ethanol metabolism together with ADH1 or compensating for its reduced contribution (Haseba et al., 2003). In addition, it was suggested that chronic binge drinking might shift the key metabolic pathway from ADH1 to ADH3 (Haseba and Ohno, 2010), therefore attributing ADH3 a more critical role at high ethanol concentrations. Notably, 4-MP inhibits ADH1 but not ADH3 (Haseba and Ohno, 1997).

Besides the involvement of ADH, also other pathways may play a critical role in ethanol metabolism in the liver (Takagi et al., 1986; Zimatkin et al., 2006; Hipólito et al., 2007; Haseba and Ohno, 2010; Figure 1). In this regard, and after decades of attempts aimed at identifying the enzyme(s) responsible for the ADH1-independent fraction of ethanol metabolism, the research has focused on the microsomal (MEOS, mostly CYP2E1; Lieber and DeCarli, 1972; Takagi et al., 1986; Teschke and Gellert, 1986) and catalase-hydrogen peroxide (H_2_O_2_; Aragon et al., 1985; Handler and Thurman, 1988b; Aragon and Amit, 1992; Bradford et al., 1993a,b; Lieber, 2004) ethanol oxidizing systems, as the ones that may come into account especially when blood alcohol is high or when drinking is chronic (Sánchez-Catalán et al., 2008). Indeed, the induction of MEOS activity due to chronic ethanol consumption seems to explain the accelerated rate of ethanol metabolism observed in chronic drinkers (Pikkarainen and Lieber, 1980). Moreover, catalase and MEOS exhibit ethanol-oxidizing activities higher than that of ADH1 and both are insensitive to pyrazoles *in vitro* (Lieber and DeCarli, 1972). Interestingly, Takagi et al. (1986) have shown that 4-MP actually inhibits MEOS in deermice genetically lacking ADH, both *in vivo* and in *in vitro* concluding that MEOS plays a significant role in ethanol oxidation. However, the role of MEOS and catalase in the consequences of systemic ethanol metabolism are yet to be fully characterized (Hipólito et al., 2007) whereas the contribution of ADH to peripheral ethanol metabolism may have been overestimated, based on some experiments where the treatment with 4-MP caused in man a very low rate of ethanol elimination (Blomstrand and Theorell, 1970), bringing to the conclusion that catalase and MEOS, and not only ADH, may be mainly responsible for ethanol’s metabolism (Blomstrand et al., 1973).

The rate-limiting step in the catalase-dependent peroxidation of ethanol is that of H_2_O_2_ generation (Oshino et al., 1973) that can be originated at high rates also from fatty acids metabolism (Handler and Thurman, 1988a). In particular, ethanol metabolism in the liver is mediated predominantly by catalase-H_2_O_2_ in the fasted state (Handler and Thurman, 1988b) and it is interesting to observe that 4-MP inhibits also acyl-CoA synthase, an enzyme essential to initiate the process of fatty acid oxidation (Bradford et al., 1993a,b). Nonetheless, some authors observed that pre-treatment with catalase inhibitors does not significantly affect ethanol pharmacokinetics (bioavailability, elimination; Tampier and Mardones, 1987; Aragon et al., 1989), suggesting that catalase does not participate actively to hepatic ethanol metabolism.

Lastly, the acetaldehyde produced by the oxidation of ethanol is thereafter transformed by ALDH to acetate, which can be further metabolized through the tricarboxylic acid cycle to generate energy (Figure 1). ALDH, that plays an important role for determining the peripheral acetaldehyde levels, is further classified into two subcategories: ALDH1 present in the cytosol and ALDH2 present in the mitochondria (Weiner and Wang, 1994). Accordingly, high acetate but not acetaldehyde concentrations can be detected in human plasma after ethanol intake (Hernández et al., 2016). Notably, eastern Asians, because of the high prevalence of ALDH2*2 allele among those populations, may be more susceptible to the effect of ethanol (and acetaldehyde) with important public health implications that may be utilized to promote ethanol abstinence or reduce ethanol consumption.

## Central Generation of Acetaldehyde

**Figure 2:**
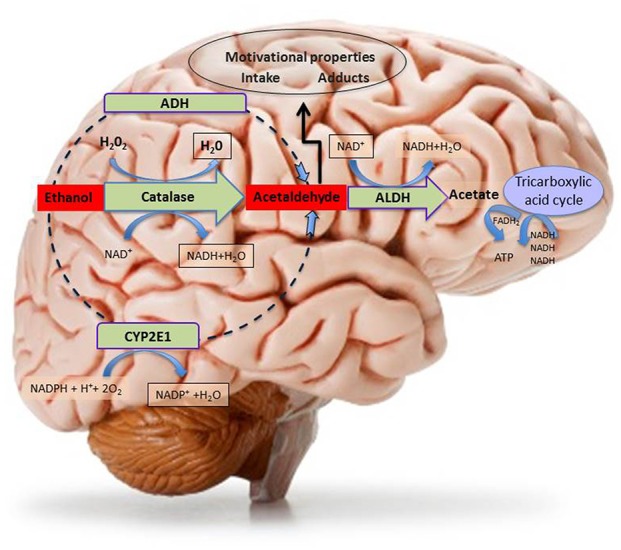
**Schematic representation of central metabolism of ethanol.** The figure depicts the three main central metabolic pathways of ethanol oxidative metabolism to acetaldehyde and the main metabolic pathways of ethanol by-product (acetaldehyde and acetate) disposal, with indication of the relative co-factors involved. Abbreviations: ADH, Alcohol dehydrogenase; ALDH, Aldehyde dehydrogenase; ATP, adenosine triphosphate; CYP2E1, isoform 2E1 of cytochrome P_450_; FADH_2_, flavin-adenine dinucleotide in its reduced form; H_2_O_2_, Hydrogen peroxide; NAD^+^, nicotinamide adenine dinucleotide coenzyme; NADPH, Nicotinamide Adenine Dinucleotide Phosphate coenzyme in its reduced form.

Ethanol can easily cross the blood-brain barrier and be metabolized in the brain. However, the cellular types the blood-brain barrier is made of, endothelial cells and oligodendrocites, highly express ALDH (Zimatkin, 1991; Zimatkin et al., 1992), which metabolizes acetaldehyde to acetate, preventing the entrance of peripherally generated acetaldehyde into the brain (Eriksson and Sippel, 1977; Deitrich et al., 1978; Hipólito et al., 2007). Thus, unless the blood-brain (metabolic) barrier activity undergoes saturation (Westcott et al., 1980; Hoover and Brien, 1981; Zimatkin, 1991), acetaldehyde levels following acute ethanol administration may hardly reach the blood concentration critical to allow acetaldehyde crossing it (Tabakoff et al., 1976; Eriksson and Fukunaga, 1993) and, therefore, affecting its targets within the CNS. Otherwise, the oxidation of ethanol to acetaldehyde can occur in the brain through pathways that involve catalase, CYP2E1 and ADH (Hipólito et al., 2007; Figure 2). In particular, although under appropriate conditions the latter seems to represent a main pathway of ethanol metabolism in the liver, it has been attributed a minor contribution in the brain as indicated by biochemical (Zimatkin et al., 2006) and behavioral studies (Escarabajal and Aragon, 2002). Interestingly, a recent study, showed that ADH, whose several isoforms, such as ADH1, 3 and 4, have been found in the mammal brain (Boleda et al., 1989; Galter et al., 2003; Hipólito et al., 2007), is related to the enhancement of voluntary ethanol intake in University of Chile Bibulous (UChB) rats, bred for their high alcohol preference, after an injection into the ventral tegmental area (VTA) of a lentiviral vector encoding for ADH (Karahanian et al., 2011). Conversely, an injection into the VTA of a lentiviral vector encoding the anti-catalase short hairpin RNA (shRNA) abolished the voluntary consumption of ethanol (Karahanian et al., 2011).

Most of *in vivo* brain acetaldehyde production depends on catalase-H_2_O_2_ peroxidase activity (Zimatkin and Buben, 2007) and catalase seems to be expressed in all neural cells (Hipólito et al., 2007) although catalase-positive staining, resulting from immunohistochemical studies, was particularly prominent in brain areas containing aminergic neuronal bodies (Zimatkin and Lindros, 1996). Accordingly, catalase mRNA was found in a large number of neurons throughout the rat brain (Schad et al., 2003). Indeed, catalase and, to a lesser extent CYP2E1, are the main pathways of central ethanol metabolism (Aragon et al., 1992; Aragon and Amit, 1993; Zimatkin et al., 2006; Hipólito et al., 2007, 2009; Sánchez-Catalán et al., 2008), as can be observed in rodent studies, showing that inhibitors of catalase, prevent the production of acetaldehyde (Aragon and Amit, 1992; Koechling and Amit, 1994).

Strong support to the evidence of the critical role of catalase-mediated metabolism of ethanol in its central effects was originally brought by a number of seminal studies by Aragon et al. (1985, 1989, 1992) and Aragon and Amit (1993). These authors reported the ability of catalase to oxidase ethanol in brain homogenates. They also showed the ability of 3-amino-1,2,4-triazole (3-AT), a catalase inhibitor, to prevent ethanol metabolism in these homogenates, both when directly applied to them (Aragon and Amit, 1992) and when previously administered *in vivo* to rats before homogenates preparation (Aragon and Amit, 1992). Behavioral studies further confirmed the suggestion of the critical role played by catalase in the mediation of some central effects of ethanol by showing that its potentiation (by acute lead acetate; Correa et al., 1999a) and its inhibition (by chronic lead acetate; Correa et al., 1999b) could increase and reduce, respectively, ethanol-induced locomotor activity. Behavioral studies, in addition, directly challenged the hypothesis of catalase-dependent production of brain acetaldehyde as a possible mediator of the psychopharmacological effects of ethanol. These studies showed that local intra-arcuate nucleus of the hypothalamus administration of another catalase inhibitor, sodium azide, could prevent the locomotor stimulating properties of ethanol (Sanchis-Segura et al., 2005) and that the systemic administration of catalase inhibitors could prevent both the locomotor stimulant effects of intra-substantia nigra pars reticulata (SNr) administration of ethanol (Arizzi-LaFrance et al., 2006) and the anxiolytic effects of systemically-administered ethanol (Correa et al., 2008). Further recent evidence on the role of catalase-mediated metabolism of ethanol was provided by the studies on ethanol-elicited locomotor stimulation, CPP (Ledesma and Aragon, 2012; Ledesma et al., 2012, 2013) and acquisition of ethanol oral self-administration (Peana et al., 2015). In summary, although acetaldehyde is generated locally in pharmacologically-significant amounts (Deng and Deitrich, 2008) by brain catalase, this process seems circumscribed to some specific brain nuclei (such as hypothalamus and midbrain) providing anatomical validation to the high behavioral specificity of the effects of drugs able to interfere with its enzymatic activity (Smith et al., 1997; Sanchis-Segura et al., 2005; Arizzi-LaFrance et al., 2006).

Overall, these studies support the hypothesis that brain-generated acetaldehyde promotes locomotor stimulation, CPP and ethanol drinking. Indeed, in order to increase acetaldehyde levels, cyanamide, an inhibitor of ALDH, has been suitably utilized locally in the VTA, in *in vivo* experiments in the presence of an otherwise ineffective concentration of ethanol on locomotor stimulation (Martí-Prats et al., 2013) and, upon systemic administration of ethanol, in order to increase the acetaldehyde’s yield in striatal microdialysates (Jamal et al., 2007). In agreement with this idea, the administration of the ALDH2-coding vector to rats bred for their alcohol preference, decreased chronic ethanol consumption demonstrating that endowing the VTA with an augmented ability to degrade acetaldehyde greatly decreases ethanol intake (Karahanian et al., 2015). Finally, acetaldehyde oxidation is required for detoxification and it can be metabolized into acetate by ALDH that plays a crucial role in further oxidizing ethanol-derived acetaldehyde (Zimatkin et al., 2006). Lastly, the acetate produced by ALDH is metabolized through the Krebs cycle to produce energy or to provide intermediates for other molecules (Hernández et al., 2016; Figure 2).

The cytochrome P450 enzymes (CYP2E1) that are involved in ethanol metabolism in the liver have also been implicated in its metabolism, in particular, in mesencephalic tyrosine hydroxylase-positive neurons (Watts et al., 1998) by reducing molecular oxygen to water and thus oxidizing ethanol to acetaldehyde (Figure 2). Notably, the induction of CYP2E1 expression by chronic ethanol treatment has been reported in a number of brain structures including the hippocampus, cerebellum and brainstem (Zhong et al., 2012). Some authors have presented solid data suggesting the role of CYP2E1 in ethanol brain metabolism, since acetaldehyde production was decreased in mouse brain homogenates from mice with CYP2E1 genetic deficiency (Quertemont et al., 2005). Furthermore, the incubation with ethanol of brain microsomes from CYP2E1 deficient mice, results in lower levels of acetaldehyde, as compared to normal mice (Vasiliou et al., 2006), although compensatory mechanisms due to increased catalase expression in these animals should be taken into account while evaluating *in vivo* the apparent lack of effects of ethanol as compared to wild type mice (Correa et al., 2009). Moreover, as mentioned above, the expression of this enzyme is induced in response to chronic drinking (Hipólito et al., 2007; Sánchez-Catalán et al., 2008) and it may thus contribute to the increased rates of ethanol elimination in heavy drinkers (Hernández et al., 2016).

## Neurobiological Effects of Ethanol and Role of Acetaldehyde

The research on the role of peripherally produced acetaldehyde in at least some of the central effects of ethanol provided different contributions in support of the suggestion that both ethanol on its own, and as ethanol-derived acetaldehyde, play a critical role in the reinforcing properties of ethanol. These investigations have been performed, in naïve rodents, after intragastric acetaldehyde or ethanol (passive) administration on CPP as well as on oral (operant) self-administration studies. In CPP experiments the inhibition of ADH1 (Peana et al., 2008) or catalase (Font et al., 2008) as well as the reduction of acetaldehyde bioavailability, by the use of sequestering agents, impairs the acquisition of ethanol-elicited CPP (Peana et al., 2008, 2009; Ledesma et al., 2013). Furthermore, acetaldehyde itself elicits the acquisition of CPP (Spina et al., 2010) through the activation of extracellular signal regulated kinase pathway via a DA D_1_ receptor-mediated mechanism (Ibba et al., 2009; Spina et al., 2010; Vinci et al., 2010). In operant experiments, acetaldehyde was reported to be orally self-administered and, similarly to ethanol, its oral self-administration was reported to be prevented by L-cysteine (Peana et al., 2010a), an agent that acts either as radical scavenger or as precursor of cysteine and that is also able to sequestrate acetaldehyde either peripherally or centrally. The operant oral self-administration paradigm is a preclinical model, in which animals are trained to emit a specific response for gaining the drug reinforcement (Grant and Samson, 1986; Samson et al., 1988). By these experiments, it was shown that the intraperitoneal administration of alpha lipoic acid, a radical scavenger that interferes with catalase-H_2_O_2_ activity (Ledesma et al., 2012), decreases maintenance, reinstatement and progressive ratio of oral operant ethanol self-administration. Likewise, L-cysteine acts also during the acquisition phase of ethanol and acetaldehyde self-administration (Peana et al., 2012, 2013b) with the acetaldehyde-binding property of cysteine being probably responsible for these effects. Likewise, Peana et al. (2015) have reported that D-penicillamine, a synthetic amino acid that strongly binds acetaldehyde, inhibits the acquisition of oral ethanol self-administration. Nonetheless, Quintanilla et al. (2016) showed that N-acetyl cysteine, a pro-drug of cysteine, fails to influence the acquisition of voluntary ethanol intake in adult female UChB rats, but greatly inhibits chronic ethanol intake. These data overall suggest that N-acetyl cysteine and L-cysteine may act by different mechanisms on the acquisition and maintenance of ethanol intake at least in part depending on experimental paradigms.

A rapidly growing body of evidence on the efficacy of radical scavengers and antioxidants such as L-cysteine (Peana et al., 2010a, 2012, 2013b), alpha lipoic acid (Ledesma et al., 2012; Peana et al., 2013a) and N-acetyl cysteine (Quintanilla et al., 2016), both on CPP experiments and in different phases (not only during the acquisition) of operant self-administration as well as on voluntary ethanol intake could be referred to the observation that a neuro-inflammatory process could be responsible of ethanol excessive taking (Montesinos et al., 2016). Indeed, the metabolism of ethanol into acetaldehyde and acetate is associated to the production of ROS that accentuate the oxidative state of cells promoting oxidative damage, neuronal injury and neurodegeneration. The oxidative balance is the result of the amount of accumulated ROS and of the activity of antioxidant enzymes. When the oxidative balance is disturbed, oxidative stress develops affecting the cell as a whole, as well as proteins, lipids and DNA. However, several defense mechanisms for reducing the deleterious effects of oxidative stress exist, and e.g., if cellular defense and repair processes fail, oxidatively damaged proteins can undergo proteasome-mediated protein degradation (Bence et al., 2001). Specifically, ethanol metabolism up-regulates the production of ROS and nitric oxide in primary cortical neurons causing blood-brain barrier dysfunction (Haorah et al., 2005, 2007, 2008). Chronic ethanol exposure has also been associated with proteasome inhibition which seems to be a key player in epigenetic mechanisms underlying alcoholism by promoting the accumulation of oxidatively damaged histones (Bardag-Gorce, 2009). Accordingly, recent findings showed that ethanol exposure reduced intracellular 20 S proteasome chymotrypsin-like activity in SH-SY5Y cells (Caputi et al., 2016) in agreement with findings obtained in the liver and the brain demonstrating that ethanol exposure decreased proteasome activity by interfering with 20 S CP and 19 S RP assembly (Bardag-Gorce, 2009; Donohue and Thomes, 2014; Erdozain et al., 2014). In addition, the CYP2E1 isoform fulfills an important role in the generation of ROS and exposure to ethanol is related to their accumulation, which may be associated to the induction of CYP2E1 in rat brain homogenates (Zhong et al., 2012). Moreover, although ALDH activity has beneficial effects, i.e., is responsible of the reduction of acetaldehyde, it also produces free radicals (Hernández et al., 2016). Notably, in the last decade, new insights into the mechanisms of the immune system response have driven research toward understanding the relationship between ethanol intake, the immune system dysregulation and its contribution in a wide range of disorders associated to ethanol exposure (Szabo and Saha, 2015), including neuro-inflammation and CNS dysfunctions (Crews et al., 2015; Montesinos et al., 2016). In this regard, it is noteworthy that antioxidant activity of human serum of patients with a diagnosis of alcohol dependence syndrome is lower than that of healthy donors (control group; Plotnikov et al., 2016).

Neuro-inflammation is associated with alcohol use disorders. Accordingly, recent work showed that the toll-like receptor 4 (TLR4) is involved in the induction of cytokines and chemokines, which promote neuro-inflammation, brain damage, behavioral and cognitive dysfunction (Pascual et al., 2015); however, it is important to note that while a selective inhibitor of these receptors was reported to decrease ethanol drinking in both ethanol-dependent and non-dependent mice (Bajo et al., 2016), other studies suggested recently that TLR4 may not be directly involved in the regulation of excessive drinking (Harris et al., 2017). Interestingly, anti-inflammatory mechanisms might also be evoked to interpret our recent observations that ethanol self-administration in Wistar rats (Peana et al., 2014) and ethanol-elicited CPP in CD-1 mice (Spina et al., 2015) were prevented by the administration of the standardized extract of the roots of *Withania somnifera* a medicinal plant renowned for its anti-inflammatory and free radical scavenger properties (Dar et al., 2015). On this line of evidence, it is worth mentioning that mesenchymal stem cells, known to reduce oxidative stress (Valle-Prieto and Conget, 2010) and to secrete anti-inflammatory cytokines (Lee et al., 2016), were able to inhibit relapse-like drinking after intracerebral administration (Israel et al., 2017).

On the other hand, chronic ethanol intake (maintenance phase of the self-administration protocols) seems to become independent of the early acetaldehyde mediated reinforcing mechanisms (Peana et al., 2015) responsible for the *first hit* (Israel et al., 2015). In this regard, the observation that the pharmacological manipulation of ethanol metabolism, by inhibition of catalase or by reduction of acetaldehyde bioavailability, does not interfere with the perpetuation (maintenance) of ethanol self-administration appears in agreement with data reported by Israel et al. (2015). To interpret these results, it was suggested that acetaldehyde could indirectly contribute to the maintenance phase of oral ethanol self-administration by the combination of two mechanisms: the first, indirect one, was hypothesized to be due to the lack of acetaldehyde itself that would make the animals to further seek and take ethanol; the second mechanism, on the other hand, was suggested (Peana et al., 2015) to be based on the decreased metabolism of ethanol that would make available its non-metabolized fraction to act onto GABA_A_ receptors resulting in further maintaining of ethanol self-administration. Otherwise, the alcohol relapse model based on the alcohol deprivation effect has also been widely used to assess ethanol craving and relapse. Thus, using this preclinical model, a recently published study showed that chemical inactivation of acetaldehyde, by D-penicillamine treatment in long-term ethanol experienced rats, prevents relapse into ethanol taking (Orrico et al., 2013). Moreover, the combined *therapy* of naltrexone and D-penicillamine prevents the delayed increase in ethanol consumption observed after continuous μ opioid (MOP) receptors blockade, suggesting the suitability of this combination as anti-relapse preclinical treatment (Orrico et al., 2014) and highlighting the role of acetaldehyde in the effects of ethanol. As also discussed in this review, a large number of neuropharmacological studies pointed to multiple neurochemical systems involved in the reinforcing effects of ethanol and to the interactions between ethanol and the CNS opioid signaling system, in particular (Coonfield et al., 2002; Oswald and Wand, 2004; Ghozland et al., 2005; Koob and Le Moal, 2006; Vukojević et al., 2008). Similarly, in human neuroblastoma SH-SY5Y cells, changes in the expression of the opioid receptors and the precursors of the opioid peptide ligands were observed in response to ethanol or acetaldehyde 40 mM and 0.4 mM, respectively (D’Addario et al., 2008), in agreement with the evidence that at least some of the neurochemical effects of ethanol are mediated by its first metabolite, and that, accordingly, ethanol must be metabolized into acetaldehyde to generate reward and reinforcement (Karahanian et al., 2011; Correa et al., 2012; Peana and Acquas, 2013). Furthermore, these data corroborate the hypothesis that the changes observed in the pro-enkephalin and k opioid receptors expression upon ethanol application are probably due to the action of his metabolite (D’Addario et al., 2008). In the meantime other studies correlated the epigenetic modifications with up/down regulation of genes caused by ethanol or acetaldehyde in the liver and rat brain tissue (Kim and Shukla, 2006; Shukla and Aroor, 2006; Shukla et al., 2007; Pandey et al., 2008). Notably, a temporal relationship between histone modifications and pro-dynorphin gene expression down-regulation was observed in a study conducted in human neuroblastoma cells (D’Addario et al., 2011) highlighting that ethanol or acetaldehyde exposure influences epigenetic regulation through histone acetylation, hence regulating propensity for DNA transcription at specific portions of the genome.

Ethanol and its metabolites may interfere with many biological processes, including neuronal differentiation, leading to severe brain damage and neurological disorders. In fact, the most severe ethanol-related damage, associated with a loss of neurons, was found following acute prenatal (Flentke et al., 2014) or chronic (Fernandes et al., 2002; Moulder et al., 2002; Soscia et al., 2006) pre- and post-natal exposure. Moreover, the generation of new neurons and their functional integration into the CNS is reported to be altered by ethanol (Nixon and Crews, 2002; Crews et al., 2006). In this regard, ethanol is known to affect cellular development interfering with brain-derived neurotrophic factor (BDNF; Climent et al., 2002; Sakai et al., 2005) and other neurotrophins (Moore et al., 2004; Bruns and Miller, 2007; Mooney and Miller, 2011). Studies conducted over the last decade by using *in vitro* models have enriched the information about this interplay. Thus, Hellmann et al. (2009) demonstrated that chronic ethanol exposure impaired neuronal differentiation of neuroblastoma cells and consistently impaired BDNF-mediated activation of the Mitogen-Activated Protein Kinase/Extracellular signal-Regulated Kinase (MAPK/ERK) cascade.

Together with phosphoinositide 3-kinase/protein kinase B (PI3K/AKT) pathway, MAPK/ERK cascade is activated by the growth factor midkine (MDK; Stoica et al., 2002), thus promoting cell survival and proliferation (Reiff et al., 2011). It has been hypothesized that MDK might be an ethanol-responsive gene since it protects against neuronal damage and neuro-degeneration (Herradón and Pérez-García, 2014) and might regulate sensitivity to ethanol (Lasek et al., 2011). In support of this possibility, MDK expression is high in the prefrontal cortex of human alcoholics (Flatscher-Bader et al., 2005; Flatscher-Bader and Wilce, 2008), and is also elevated in the brains of mice genetically predisposed to consume high amounts of ethanol (Mulligan et al., 2006). Based on this evidence, it has been hypothesized that ethanol engages MDK and the activation of its receptor, the anaplastic lymphoma kinase (ALK). Intriguing studies, employing the neuroblastoma SH-SY5Y and IMR-32 cell lines, demonstrated that MDK and ALK are ethanol-responsive and that the activation of ALK signaling by ethanol is dependent on MDK expression. In fact, not only ethanol increased MDK and ALK gene expression, but also caused a rapid increase in the phosphorylation of ALK and ERK (He et al., 2015).

## Role of Brain Ethanol-Derived Acetaldehyde in The Effects of Ethanol

Acetaldehyde is a neuropharmacologically active substance, which has reinforcing properties *on its own* and provokes, although at lower doses/concentrations than ethanol, some behavioral responses similar to its parent compound suggestive of the fact that it could be responsible of some of its effects (Correa et al., 2012). Several authors have shown that the intracerebral administration of acetaldehyde induces locomotor stimulation (Correa et al., 2003, 2009; Arizzi-LaFrance et al., 2006; Sánchez-Catalán et al., 2009), CPP (Smith et al., 1984; Quertemont and De Witte, 2001; Quintanilla et al., 2002), conditioned taste preference (Brown et al., 1978) and self-administration (Brown et al., 1980; Myers et al., 1982; Rodd-Henricks et al., 2002). Likewise, microinjections of acetaldehyde into the posterior VTA (pVTA) increase DA release in the shell of the nucleus accumbens (Acb), measured by microdyalisis (Melis et al., 2007; Deehan et al., 2013). Moreover, electrophysiological studies have demonstrated that acetaldehyde administration stimulates the activity of VTA DA neurons *in vitro* (Melis et al., 2007; Diana et al., 2008) and *in vivo* (Foddai et al., 2004; Enrico et al., 2009). However, although highly suggestive, all the above evidence does not represent unequivocal proof that acetaldehyde is the key element for the development of the neurobiological effects of ethanol (Correa et al., 2012; Israel et al., 2015).

In order to address this critical question, and to assess the role of brain ethanol-derived acetaldehyde, the strategy more commonly used has been to evaluate the consequences of the modulation of the enzymatic systems involved in brain ethanol metabolism (Hipólito et al., 2007). Indeed, the catalase activity has been the most targeted one, due to its high contribution to brain ethanol metabolism. Thus, a decreased activity of brain catalase would elicit a reduction or blockade of ethanol effects, as a consequence of decreased levels of acetaldehyde formation. Arizzi-LaFrance et al. (2006) demonstrated that the locomotor activation induced by ethanol microinjection into substantia nigra pars compacta (SNc) could be blocked with the systemic administration of sodium azide. Otherwise, intra-cerebral injection of sodium azide is able to prevent the locomotor-stimulating effects of ethanol microinjection into the hypothalamic arcuate nucleus (Pastor and Aragon, 2008) and the decreased locomotion induced by intraperitoneal administration of ethanol in rats (Sanchis-Segura et al., 2005). Moreover, the direct correlation between voluntary consumption of ethanol and brain catalase activity (Amit and Aragon, 1988; Gill et al., 1996) highlights the role of brain acetaldehyde formation. Consistent with this idea, the intra-VTA microinjection of a lentiviral vector encoding a shRNA for silencing catalase gene expression and reducing the catalase content has been shown to decrease the voluntary ethanol consumption and alcohol deprivation effect (Karahanian et al., 2011; Tampier et al., 2013). Similarly, brain ALDH activity has been modulated to increase the locomotor-stimulating effects of ethanol in rats (Spivak et al., 1987; Martí-Prats et al., 2013). Moreover, a positive correlation between voluntary ethanol consumption and brain ALDH activity (Sinclair and Lindros, 1981; Socaransky et al., 1984) has been described, also, by means of a lentiviral vector encoding for ALDH and enhancing acetaldehyde metabolism (Karahanian et al., 2015). Besides the evidence gathered by behavioral studies, the involvement of local acetaldehyde formation in the central effects of ethanol was also provided by an *in vitro* electrophysiological approach showing that local catalase inhibition, through 3-AT administration, prevents the effect of ethanol on pVTA DA neurons (Melis et al., 2007).

Several recent studies have used the acetaldehyde-sequestering agent, D-penicillamine, to assess the impact of the reduction of brain acetaldehyde levels on the development of the psychopharmacological effects of ethanol. D-penicillamine is an amino acid that interacts with acetaldehyde to form a stable adduct (Nagasawa et al., 1980), which has been detected in plasma, liver and brain following ethanol administration (Serrano et al., 2007). Moreover, intra-cisternal administration of D-penicillamine was shown to prevent the locomotor stimulation and CPP produced by intra-cisternal ethanol administration in newborn rats (Pautassi et al., 2011; March et al., 2013), whereas the intra-cerebroventricular pretreatment with D-penicillamine was reported to decrease the voluntary ethanol intake in rats (Font et al., 2006). In addition, more direct than previously described evidence has pointed to the VTA as a key brain region for the effects of ethanol-derived acetaldehyde following sequestering-agents pretreatment since the intra-VTA administration of D-penicillamine prevents the alcohol deprivation effect in Wistar rats (Orrico et al., 2013).

While the above described studies confirm that *in situ* acetaldehyde formation from ethanol is necessary to the development of the psychopharmacological effects of ethanol, the mechanism through which ethanol or acetaldehyde activate VTA DA neurons, and therefore the mesolimbic DA system, is not yet fully understood. Nowadays, it has been shown that ethanol modulates the activity of DA neurons through its interaction with several neurochemical and neuroendocrine systems including GABA, glutamate, opioid, cannabinoid and corticotropin-releasing factor systems and cytoplasmic elements (Gessa et al., 1985; Morikawa and Morrisett, 2010; D’Addario et al., 2013; Erdozain and Callado, 2014). Both ethanol and acetaldehyde are able to stimulate DA neurons through ion channels modulation (Brodie et al., 1990; Okamoto et al., 2006; Melis et al., 2007), and it has been shown, by electrophysiological approaches, that prevention of acetaldehyde formation or its inactivation leads to blockade of ethanol effects (Foddai et al., 2004; Melis et al., 2007; Enrico et al., 2009).

In particular, as to the endogenous opioid system, there is a general agreement that it is involved in the neurobiological effects of ethanol and a number of hypotheses have been proposed to explain how this interaction may take place. Several evidences support the notion that the activation of MOP receptors expressed onto VTA GABA neurons and other GABA afferents (Johnson and North, 1992; Jalabert et al., 2011; Sánchez-Catalán et al., 2014), is critically involved in the stimulation of VTA DA neurons after ethanol or acetaldehyde (Mereu and Gessa, 1985; Xiao et al., 2007; Fois and Diana, 2016). Consistent with this idea, behavioral studies have shown that the microinjection of naltrexone or β-funaltrexamine (an irreversible MOP receptor antagonist) can prevent the motor activation induced by the intra-pVTA administration of ethanol or acetaldehyde (Sánchez-Catalán et al., 2009). Moreover, such preventive effect has been also observed by using electrophysiological approaches (Xiao and Ye, 2008; Guan and Ye, 2010; Fois and Diana, 2016).

**Figure 3:**
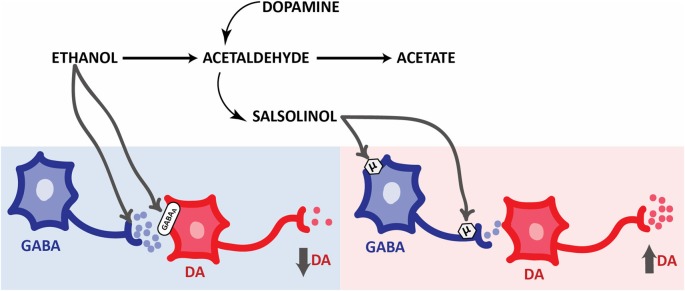
**Opposite responses elicited by ethanol and its derivatives on the activity of mesolimbic dopaminergic system.** Simplified schematic representation of the effects of ethanol metabolites on pVTA DA neurons. Ethanol evokes an inhibition of pVTA DA neurons through presynaptic (Weiner and Valenzuela, 2006) and postsynaptic GABAergic mechanisms. On the other hand, salsolinol induces an excitation of pVTA DA neurons through MOP receptors activation located in the soma and terminals of GABA neurons. Abbreviations: DA, dopamine. GABA, γ-aminobutiric acid. GABA_A_, GABA receptors type A. μ, MOP receptors.

Besides the stimulating effect of ethanol or acetaldehyde into the pVTA, which seems to involve the MOP receptors and, ultimately, ethanol by-products (see below), it has been observed that ethanol on its own can activate VTA GABA neurons, eliciting inhibition of DA neurons (Steffensen et al., 2009). The reinforcing properties of ethanol would depend on the action of ethanol itself and of its metabolites, as proposed by Martí-Prats et al. (2013). Indeed, these authors demonstrated that the intra-pVTA administration of a low dose of ethanol did not induce any locomotor effect in rats, which implies a balanced effect between those of ethanol and those of its metabolites. Thus, decreased levels of ethanol-derived acetaldehyde by catalase inhibition (sodium azide) or by acetaldehyde inactivation (D-penicillamine), converts an inactive ethanol dose into a depressive one. On the contrary, the increase of ethanol-derived acetaldehyde levels obtained by ALDH inhibition (cyanamide), converts the ethanol dose into a stimulating one. Thereby, the decrease of the ethanol-metabolized fraction (acetaldehyde and, perhaps, salsolinol) discloses the inhibitory action of the non-metabolized fraction of ethanol, whereas the increase of the metabolized fraction discloses a stimulatory effect. Following this elegant approach, Martí-Prats et al. (2015) developed further research in order to disentangle the mechanism of action of *such* ethanol non-metabolized fraction. Accordingly, GABA_A_ antagonism (bicuculline) was able to convert an inactive ethanol dose into an active one. Moreover, bicuculline administration was also able to prevent the motor depression observed after D-penicillamine pretreatment (therefore, no motor effect was observed; Martí-Prats et al., 2013). On the other hand, the intra-pVTA administration of β-funaltrexamine induced a decrease of motor activity of the animals treated with an inactive dose of ethanol, suggesting that the consequence of MOP receptor blockade on this effect could be attributable to the blockade of the effects of the ethanol-metabolized fraction (Martí-Prats et al., 2015; Figure 3).

Overall, these studies provide evidence of the key role of ethanol-derived acetaldehyde, formed *in situ*, in the mechanisms underlying the psychopharmacological effects of ethanol. However, it is noteworthy that the involvement of other by-products of ethanol metabolism such as salsolinol are also a key issue to the neurobiological basis of these effects, as will be discussed in the next section.

## Beyond Acetaldehyde: Role of Salsolinol

Acetaldehyde is a highly reactive compound with a very short half-life and reacts with biogenic amines to produce condensation products known as tetrahydroisoquinolines (THIQs). In the 70s, several investigators proposed the *THIQs theory of alcoholism*, pointing at these molecules as possible mediators of part of the ethanol effects on the mesolimbic system and consequently as responsible of playing an important role in alcoholism (Cohen and Collins, 1970; Davis and Walsh, 1970; Melchior and Myers, 1977; Duncan and Deitrich, 1980). Although in the late 70s and 80s, some investigators explored the role of some THIQs, including betacarbolines and tetrahydropapaveroline, on ethanol intake and DA release (Myers and Melchior, 1977; Tuomisto et al., 1982; Airaksinen et al., 1983; Myers and Robinson, 1999), no more studies have increased our knowledge in their role in alcoholism. Otherwise, the condensation product of acetaldehyde with DA, salsolinol (1-methyl-6,7-dihidroxy-1,2,3,4-tetrahydroisoquinoline, for detailed review on salsolinol formation see Hipólito et al., 2012), has been specially investigated trying to clarify its role in the neurobiological basis of alcohol addiction (Figure 3). When investigating the intriguing relationship between ethanol and salsolinol four main questions arise: (i) is salsolinol produced in pharmacologically significant concentrations after ethanol intake?; (ii) does salsolinol activate the DA mesolimbic pathway to induce alcohol use disorders -related behaviors?; if so, (iii) how is salsolinol activating this system?; and (iv) is salsolinol necessary for ethanol to exert its activating effect on the dopaminergic mesolimbic system?

As to the first question, the issue of salsolinol formation in the brain after ethanol administration is still unclear. In fact, beginning from the first evidences of salsolinol formation in the brain of rodents after ethanol administration brought in the mid 70 by Collins and Bigdeli (1975), different laboratories have obtained controversial data. Actually, some of these reports show an increase of salsolinol levels (Myers et al., 1985; Matsubara et al., 1987; Jamal et al., 2003a,b,c; Starkey et al., 2006; Rojkovicova et al., 2008) while others show no alterations in the brain after different protocols of ethanol administration (O’Neill and Rahwan, 1977; Baum et al., 1999; Haber et al., 1999; Lee et al., 2010). Moreover, the investigations reporting increased brain salsolinol upon chronic ethanol intake or chronic ethanol administration, were performed in rats allowed to access *ad libitum* lab chow either before and throughout the study (Sjöquist et al., 1982). Interestingly, when such rat studies were repeated using chronic ethanol intake in DOPA- and salsolinol-free liquid diets, no changes were found of endogenous brain salsolinol concentrations (Collins et al., 1990). However, when the ethanol-liquid diets were supplemented with DOPA, salsolinol levels were raised (Collins et al., 1990) suggesting that, upon prolonged ethanol intake, elevations of endogenous salsolinol concentrations, as well as of those of other THIQs, seem to depend on a number of factors including the brain region investigated, the duration of intake and the associated dietary constituents (Lee et al., 2010). The differences observed in the data from these studies have been extensively summarized and analyzed in a previous review on this specific topic (Hipólito et al., 2012). Notably, in this regard, only one report, in the last 5 years has added more knowledge to this issue. In this study, authors detected an increase in salsolinol levels in fetal rat brain after an alcoholization protocol from GD8 to GD20 (Mao et al., 2013). However, similarly to the studies mentioned previously, there is still a lack of a direct demonstration that may provide us a precise correlation between the ethanol load and the concentration of salsolinol achieved in the brain tissue. In summary, convincing data, clearly demonstrating brain (neuronal or glial) salsolinol formation in ethanol-treated and/or ethanol-consuming animals, are still missing.

As to the second question, in spite of the uncertainties mentioned above, the latest investigations on the psychopharmacological effects of salsolinol appear in support of the *THIQs theory of alcoholism*. Throughout the last 15 years, different laboratories have successfully shown that salsolinol exerts, on the mesolimbic system, effects similar to ethanol, suggesting that at least some of the effects of ethanol may be mediated by salsolinol. These latest neurochemical and behavioral studies have shed a bit of light on the previous controversial data where an effect or a lack of salsolinol’s effect on the dopaminergic system had been reported. The most recent data, obtained with the intracerebral administration of low doses (0.3–30 μg) of salsolinol, revealed that it robustly increases motor activity and produces motor sensitization (Quintanilla et al., 2014, 2016); this is in contrast with those studies where salsolinol, administered at high doses (100–300 μg intracranial or 10–100 mg i.p.), was reported to induce no change of motor behavior (Antkiewicz-Michaluk et al., 2000a; Vetulani et al., 2001). Indeed, Hipólito et al. (2011) and Quintanilla et al. (2016) have shown that the administration of 30 pmol of salsolinol directly into the rats pVTA increases motor activity and induces motor sensitization, being R-SAL the active stereoisomer (Quintanilla et al., 2016). These data are similar, in terms of profile and activation magnitude, to the previously reported effects of intra-pVTA administration of ethanol and acetaldehyde on motor activity (see “Central Generation of Acetaldehyde” Section for discussion on this aspect and Sánchez-Catalán et al., 2009) but with the striking difference that salsolinol is ~100 times more potent than ethanol and acetaldehyde. Very interestingly, the pVTA seems also to be a key brain area for salsolinol reinforcing effects. In a first study, using the intracranial self-administration procedure (ICSA) in rats, Rodd et al. (2008) showed that salsolinol is self-administered at concentrations ranging from 0.03 to 0.3 μM/infusion directly into this brain region. Curiously these authors have used the same ICSA protocol with both ethanol and acetaldehyde as reinforcers showing that these three compounds share a similar response profile in this protocol but with different potencies (being salsolinol ≥ acetaldehyde >> ethanol; Rodd et al., 2004, 2005). Furthermore, although context association learning using salsolinol as reinforcer (i.e., as an unconditioned stimulus) was already revealed in a study using its peripheral, systemic, administration in a CPP paradigm (Matsuzawa et al., 2000), we recently demonstrated the involvement of the pVTA also in this effect (Hipólito et al., 2011). In this study, in fact, the administration of 30 pmol of salsolinol (the same dose used in the motor activity studies) into the pVTA, associated to a context, increased the time spent by the rats in that context on the test day under drug free conditions (Hipólito et al., 2011). Five years after this study, other investigators have reproduced these data also showing that R-salsolinol is the active stereoisomer responsible for CPP induction (Quintanilla et al., 2016).

In accordance with the above locomotor activity, ICSA and CPP studies, the administration of similar doses of salsolinol into the pVTA increases DA release in the Acb shell (Hipólito et al., 2011; Deehan et al., 2013) through an indirect stimulation of the pVTA DA neurons by a mechanism suggested by *ex vivo* electrophysiological studies (Xie et al., 2012) that will be discussed below.

Indeed, the Acb has also been shown to be responsive to the pharmacological action of salsolinol. Thus, salsolinol is also self-administered into the Acb shell of alcohol-preferring (P) rats (Rodd et al., 2003) reaching its maximal number of responses at 3 μM, a concentration 30 times greater than that (0.1 μM) required to obtain the maximal response when self-administered into the pVTA (Rodd et al., 2008). Notably, although these two studies were performed within the same lab and following an identical ICSA procedure, before concluding that the Acb shell is less sensitive than the pVTA to the reinforcing properties of salsolinol, it is important to note that, in the Acb shell experiments, Rodd et al. (2003) used P rats, which are genetically bred to present high levels of alcohol intake.

Moreover, the neurochemical effects of salsolinol in the Acb have been characterized by *in vivo* brain microdialysis in another study (Hipólito et al., 2009). Interestingly, in this research salsolinol showed a differential effect depending on the accumbal region studied. In fact, salsolinol reduced DA levels down to 50% from baseline when delivered into the shell whereas it increased them up to 130% when administered into the core (Hipólito et al., 2009). These regional differences in response to salsolinol were previously specifically observed for MOP and δ opioid receptor agonists (Hipólito et al., 2008). In this study, the application of these agonists increased DA release in the Acb core but decreased it in the Acb shell with a profile and magnitude similar to that reported for salsolinol (Hipólito et al., 2009). In this regard, it is appropriate to note that in the ICSA experiments of Rodd et al. (2003), salsolinol was self-administered directly into the Acb shell, whereas these microdialysis data show that salsolinol is able to decrease DA release in the Acb shell. One possible explanation for this apparent discrepancy might be found in the different concentrations of salsolinol. In the case of the ICSA studies, the concentration of salsolinol able to induce the maximum number of administrations was 3 μM whereas that one able to induce the maximum decrease of DA was 5 μM, which, due to the recovery of the dialysis membrane (~10%), is notably lower than the concentration of salsolinol in the self-administered solution. Another possibility relies on the different modalities of salsolinol administration followed in these experiments: microdialysis studies, in fact, show responses (collected every 20 min) upon the acute application of salsolinol whereas ICSA studies use repeated and more prolonged applications of salsolinol (several sessions). Hence, based on the rate of salsolinol self-administration more than one dose of salsolinol would be administered in 20 min and this observation makes highly fallacious comparing studies with so different time scales and administration modalities.

The opioid system has been suggested to mediate the salsolinol (as well as the acetaldehyde, Peana et al., 2011) effects in the CNS. In a very early set of studies salsolinol showed to bind to opioid receptors and produce opioid-like effects (Tampier et al., 1977; Blum et al., 1978; Fertel et al., 1980; Lucchi et al., 1982). Based on these studies and on the structural similarities of salsolinol with the MOP receptor agonist morphine, most part of the neurobiological consequences deriving from the administration of salsolinol were shown to be impeded by pharmacological blockade of MOP receptors. In particular, the blockade in the pVTA of these receptors by the selective and irreversible MOP antagonist β-funaltrexamine impairs the salsolinol-elicited increase in motor activity (Hipólito et al., 2010), the acquisition of CPP after either its systemic (Matsuzawa et al., 2000) and intra-pVTA administration (Hipólito et al., 2010) as well as the increase in accumbal DA release elicited by intra-pVTA salsolinol administration (Hipólito et al., 2011). Moreover, although, unfortunately, there are no data on the effects of opioid antagonists on salsolinol self-administration, the pharmacology of salsolinol ICSA has been investigated by Rodd et al. (2003, 2005, 2008). In their studies, these authors explored the role of the dopaminergic (D_2_ and D_3_ subtypes) and the serotoninergic (5-HT_3_ subtype) receptors in the reinforcing properties of salsolinol. The co-infusion of the D_2_/D_3_ receptor antagonist, sulpiride, with salsolinol completely blocked the number of self-infusions into the Acb shell (Rodd et al., 2003); similarly, although by a different mechanism, co-administration of the D_2_/D_3_ receptor agonist, quinpirole, significantly reduced the number of responses on the active lever to obtain the ICSA of salsolinol into the pVTA (Rodd et al., 2008). Thus, although several studies have excluded a direct interaction of salsolinol with D_1_ and D_2_ receptors (Antkiewicz-Michaluk et al., 2000a,b; Tóth et al., 2002; Homicsko et al., 2003; Székács et al., 2007), these data indicate that somehow DA receptors are involved in the reinforcing properties of salsolinol in the mesolimbic pathway. Moreover, in similar ICSA experiments performed to deliver salsolinol into the pVTA, Rodd et al. have also investigated the implication of 5-HT_3_ receptors and showed that the application of the selective antagonist of the 5-HT_3_ receptors, ICS 205, 930, significantly diminished the number of salsolinol self-administrations. The authors of this study hypothesized that the involvement of 5-HT_3_ receptors may be indirect, based on previous reports that show an enhancement of serotonin extracellular levels after salsolinol administration in the rats striatum (Maruyama et al., 1992; Nakahara et al., 1994). Unfortunately, no more experiments have been done to test this possibility and characterize further the pharmacology of ICSA of salsolinol.

As to the third question, in a set of more direct experiments, Xie et al. (2012) tried to shed light on how salsolinol activates DA neurons of the mesolimbic pathway, by using the patch clamp technique. In these experiments, DA neurons were patched and action potentials (APs), as well as spontaneous inhibitory postsynaptic currents (sIPSC) from GABA inputs were recorded. Salsolinol was able to increase, dose-dependently, the number of APs/10 s and to reduce the number of sIPSC/10 s after its bath application at concentrations as low as 0.003–1.0 μM. These data confirm the ability of salsolinol to activate pVTA DA neurons through an indirect mechanism that involves a decrease of GABA release onto DA neurons. Furthermore, following the idea of MOP receptors involvement in the effects of salsolinol on mesolimbic DA system, the authors used a non-selective antagonist, naltrexone, and found that it impairs salsolinol effects on both the APs and the sIPSC. Taking into account the organization of the pVTA (Johnson and North, 1992), it seems reasonable to suggest that salsolinol is able to activate MOP receptors mainly located onto pVTA GABA neurons and other GABA afferents that control the DA neurons activity. As a consequence of the GABA neurotransmission inactivation, DA neurons (under tonic control from GABA neurons) may be activated through a disinhibition mechanism (Xie et al., 2012). Notably, this mechanism of action offers a reasonable mechanistic explanation of the neurochemical and behavioral experiments exposed above but also may give an explanation of how ethanol is able to interact with the opioid system to mediate its activating effects (Font et al., 2013). Nonetheless, there are still unresolved questions about salsolinol (i.e., the involvement of 5-HT_3_ receptor), and probably salsolinol mechanism of action is much more complicated. Other possibilities, besides the MOP and the 5-HT_3_ receptors, have been explored *in vitro*. After another set of electrophysiology experiments, Xie and Ye (2012) concluded that the effect of salsolinol on DA neurons involves at least three mechanisms: (i) a depolarizing action of DA neurons by itself; (ii) an activating action of MOP receptors onto GABAergic inputs; and (iii) an enhancing action of presynaptic glutamatergic transmission onto DA neurons via activation of DA D_1_ receptors on the glutamatergic terminals. However, as previously discussed, this is still an open question that will need more data from different techniques to be clarified.

The final question, perhaps the most difficult to answer and at the same time the most important one, refers to the involvement of salsolinol in the neurobiological effects of ethanol. All data discussed above give support to the *THIQs theory of alcoholism* without providing a direct confirmation of the crucial role of salsolinol in the effects of ethanol. In investigating the neurobiological effects of salsolinol, others and we always administered exogenous salsolinol to observe its effects but never used ethanol to obtain salsolinol *in situ*. Obviously, the lack of knowledge about salsolinol formation from ethanol does not allow us to manipulate the metabolism as others and we did to study acetaldehyde’s effects. Noteworthy, a recent study from Melis et al. (2015) has provided a direct evidence of salsolinol involvement in ethanol activating effects of DA neurons in the pVTA. In this very well designed electrophysiological study, frequency of APs was measured in DA depleted mice to allow the manipulation of the hypothesized steps of salsolinol formation from ethanol metabolism. The study by Melis et al. (2015) reported that ethanol could increase the frequency of APs of DA neurons in slices from DA depleted mice only if in the presence of exogenously added DA. Moreover, DA neurons from slices obtained from DA depleted mice that were treated with 3-AT (that prevents acetaldehyde’s and, consequently, salsolinol’s formation) showed no APs increases in response to ethanol. These data reveal that the formation of salsolinol from ethanol-derived acetaldehyde is necessary for the ethanol activating effects on DA neurons in the pVTA.

In conclusion, although there is a yet growing interest in the field with high expectancies of providing very critical insights on this topic, some of the questions are, unfortunately, still unanswered making the role of salsolinol in the effects of ethanol a matter of further debate in the field of alcohol addiction.

## Summary and Future Developments

As acetaldehyde is involved in many actions of ethanol in the brain, including behavioral changes and neuronal damage, the drugs used to interfere with ethanol metabolism or reduce acetaldehyde levels may represent a valuable therapy in the management of the large number of alcohol use disorders including relapse into ethanol taking and, in combination with the existing ones, might improve the outcomes of current pharmacological therapies. Moreover, compounds endowed with anti-oxidant properties represent nowadays potentially good candidates to treat distinct phases of ethanol misuse. The neuroimmune mechanisms of ethanol and acetaldehyde offer new approaches to develop more effective pharmacotherapies to treat ethanol-related neuropathologies. This recent latter evidence could explain the efficacy of different radical scavengers and antioxidant drugs in the reduction of ethanol-, ethanol-derived acetaldehyde- and acetaldehyde-dependent effects. Moreover, as some pioneering and recent studies point to the relevance of the cortico-striatal glutamatergic transmission to the development of addiction to psychostimulant and other drugs of abuse, further research appears needed to disentangle the exact relationship between ethanol, acetaldehyde and salsolinol in this pathway, which might lead to develop new treatments.

Moreover, the latest research on the acetaldehyde-DA adduct points to salsolinol as a strategic tile in the puzzle that explains the activating effects of ethanol that may eventually lead to alcohol use disorders in some individuals. In this regard, it is noteworthy that all the potential pharmacological tools, suggested by the above evidence, may be able to prevent either salsolinol formation or salsolinol actions onto DA neurons. However, although the gathered evidence discloses potential targets of promising therapies, it also requires great caution as most part, if not all, of the data discussed in the present review were obtained upon acute administrations. In other words, since in this scenario we are still missing the data under and after chronic exposure to ethanol, acetaldehyde and salsolinol, modeling more advanced states of addiction (Belin-Rauscent et al., 2016) appears the must for future research in order to really gain understanding on which may be the real role of ethanol and its by-products in abnormal ethanol taking behaviors.

In conclusion, significant new evidence supports the role of acetaldehyde and salsolinol in many actions of ethanol in the CNS which offers new insights for the search of new targets and for the discovery of effective pharmacotherapies against the development of alcohol abuse and dependence.

## Author Contributions

All authors, based on their expertise, contributed to write and critically edit this review article.

## Funding

LH funds were from the Spanish Government MICINN (PSI2016-77895-R); EA funds were from Regione Autonoma della Sardegna (RAS), CUP B71J11001480002.

## Conflict of Interest Statement

The authors declare that the research was conducted in the absence of any commercial or financial relationships that could be construed as a potential conflict of interest.

## References

[B1] Airaksinen M. M. Mähönen M. Tuomisto L. Peura P. Eriksson C. J. (1983). Tetrahydro-beta-carbolines: effect on alcohol intake in rats. Pharmacol. Biochem. Behav. 18, 525–529. 10.1016/0091-3057(83)90230-7 6634862

[B2] Amit Z. Aragon C. M. G. (1988). Catalase activity measured in rats naive to ethanol correlates with later voluntary ethanol consumption: possible evidence for a biological marker system of ethanol intake. Psychopharmacology 95, 512–515. 10.1007/bf00172965 3145522

[B3] Antkiewicz-Michaluk L. Michaluk J. Romanska I. Papla I. Vetulani J. (2000a). Antidopaminergic effects of 1,2,3,4-tetrahydroisoquinoline and salsolinol. J. Neural. Trans. 107, 1009–1019. 10.1007/s007020070049 11041279

[B4] Antkiewicz-Michaluk L. Romañska I. Papla I. Michaluk J. Bakalarz M. Vetulani J.. (2000b). Neurochemical changes induced by acute and chronic administration of 1,2,3,4-tetrahydroisoquinoline and salsolinol in dopaminergic structures of rat brain. Neuroscience 96, 59–64. 10.1016/s0306-4522(99)00533-3 10683410

[B7] Aragon C. M. G. Amit Z. (1992). The effect of 3-amino-1,2,4-triazole on voluntary ethanol consumption: evidence for brain catalase involvement in the mechanism of action. Neuropharmacology 31, 709–712. 10.1016/0028-3908(92)90150-n 1407407

[B6] Aragon C. M. G. Amit Z. (1993). Differences in ethanol-induced behaviors in normal and acatalasemic mice: systematic examination using a biobehavioral approach. Pharmacol. Biochem. Behav. 44, 547–554. 10.1016/0091-3057(93)90165-p 8451258

[B8] Aragon C. M. G. Rogan F. Amit Z. (1992). Ethanol metabolism in rat brain homogenates by a catalase-H_2_O_2_ system. Biochem. Pharmacol. 44, 93–98. 10.1016/0006-2952(92)90042-h 1632841

[B9] Aragon C. M. G. Spivak K. Amit Z. (1985). Blockade of ethanol induced conditioned taste aversion by 3-amino-1,2,4-triazole: evidence for catalase mediated synthesis of acetaldehyde in rat brain. Life Sci. 37, 2077–2084. 10.1016/0024-3205(85)90579-x 2999539

[B5] Aragon C. M. G. Spivak K. Amit Z. (1989). Effects of 3-amino-1,2,4-triazole on ethanol-induced open-field activity: evidence for brain catalase mediation of ethanol’s effects. Alcohol. Clin. Exp. Res. 13, 104–108. 10.1111/j.1530-0277.1989.tb00293.x 2646962

[B10] Arizzi-LaFrance M. N. Correa M. Aragon C. M. G. Salamone J. D. (2006). Motor stimulant effects of ethanol injected into the substantia nigra pars reticulata: importance of catalase-mediated metabolism and the role of acetaldehyde. Neuropsychopharmacology 31, 997–1008. 10.1038/sj.npp.1300849 16123765

[B503] Bagnardi V. Rota M. Botteri E. Tramacere I. Islami F. Fedirko V.. (2013). Light alcohol drinking and cancer: a meta-analysis. Ann. Oncol. 24, 301–308. 10.1093/annonc/mds337 22910838

[B11] Bajo M. Montgomery S. E. Cates L. N. Nadav T. Delucchi A. M. Cheng K.. (2016). Evaluation of TLR4 inhibitor, T5342126, in modulation of ethanol-drinking behavior in alcohol-dependent mice. Alcohol Alcohol. 51, 541–548. 10.1093/alcalc/agw026 27151970 PMC5004745

[B12] Bardag-Gorce F. (2009). Nuclear effects of ethanol-induced proteasome inhibition in liver cells. World J. Gastroenterol. 15, 1163–1167. 10.3748/wjg.15.1163 19291815 PMC2658853

[B13] Baum S. S. Hill R. Kiianmaa K. Rommelspacher H. (1999). Effect of ethanol on (R)- and (S)-salsolinol, salsoline and THP in the nucleus accumbens of AA and ANA rats. Alcohol. 18, 165–169. 10.1016/s0741-8329(98)00080-9 10456568

[B14] Belin-Rauscent A. Fouyssac M. Bonci A. Belin D. (2016). How preclinical models evolved to resemble diagnostic criteria of drug addiction. Biol. Psychiatry 79, 39–46. 10.1016/j.biopsych.2015.01.004 25747744 PMC4702261

[B15] Bence N. F. Sampat R. M. Kopito R. R. (2001). Impairment of the ubiquitin-proteasome system by protein aggregation. Science 292, 1552–1555. 10.1126/science.292.5521.1552 11375494

[B16] Blomstrand R. Kager L. Lantto O. (1973). Studies on the ethanol-induced decrease of fatty acid oxidation in rat and human liver slices. Life Sci. 13, 1131–1141. 10.1016/0024-3205(73)90380-9 4762602

[B17] Blomstrand R. Theorell H. (1970). Inhibitory effect on ethanol oxidation in man after administration of 4-methylpyrazole. Life Sci. 9, 631–640. 10.1016/0024-3205(70)90214-6 5478078

[B18] Blum K. Hamilton M. G. Hirst M. Wallace J. E. (1978). Putative role of isoquinoline alkaloids in alcoholism: a link to opiates. Alcohol. Clin. Exp. Res. 2, 113–120. 10.1111/j.1530-0277.1978.tb04710.x 350073

[B19] Boleda M. D. Julià P. Moreno A. Parés X. (1989). Role of extrahepatic alcohol dehydrogenase in rat ethanol metabolism. Arch. Biochem. Biophys. 274, 74–81. 10.1016/0003-9861(89)90416-5 2774584

[B20] Bradford B. U. Forman D. T. Thurman R. G. (1993a). 4-Methylpyrazole inhibits fatty acyl coenzyme synthase and diminishes catalase-dependent alcohol metabolism: has the contribution of alcohol dehydrogenase to alcohol metabolism been previously overestimated? Mol. Pharmacol. 43, 115–119. 8423764

[B21] Bradford B. U. Seed C. B. Handler J. A. Forman D. T. Thurman R. G. (1993b). Evidence that catalase is a major pathway of ethanol oxidation *in vivo*: dose-response studies in deer mice using methanol as a selective substrate. Arch. Biochem. Biophys. 303, 172–176. 10.1006/abbi.1993.1269 8489262

[B22] Brodie M. S. Shefner S. A. Dunwiddie T. V. (1990). Ethanol increases the firing rate of dopamine neurons of the rat ventral tegmental area *in vitro*. Brain Res. 508, 65–69. 10.1016/0006-8993(90)91118-z 2337793

[B23] Brown Z. W. Amit Z. Smith B. (1980). Intraventricular self-administration of acetaldehyde and voluntary consumption of ethanol in rats. Behav. Neural Biol. 28, 150–155. 10.1016/s0163-1047(80)91487-9 7362581

[B24] Brown Z. W. Amit Z. Smith B. Rockman G. E. (1978). Differential effects on conditioned taste aversion learning with peripherally and centrally administered acetaldehyde. Neuropharmacology 17, 931–935. 10.1016/0028-3908(78)90134-x 724100

[B25] Bruns M. B. Miller M. W. (2007). Neurotrophin ligand-receptor systems in somatosensory cortex of adult rat are affected by repeated episodes of ethanol. Exp. Neurol. 204, 680–692. 10.1016/j.expneurol.2006.12.022 17320080 PMC1995597

[B26] Caputi F. F. Carboni L. Mazza D. Candeletti S. Romualdi P. (2016). Cocaine and ethanol target 26S proteasome activity and gene expression in neuroblastoma cells. Drug Alcohol Depend. 161, 265–275. 10.1016/j.drugalcdep.2016.02.012 26922280

[B27] Chevens L. C. (1953). Antabuse addiction. Br. Med. J. 1, 1450–1451. 10.1136/bmj.1.4825.1450-c 13042308 PMC2016633

[B28] Climent E. Pascual M. Renau-Piqueras J. Guerri C. (2002). Ethanol exposure enhances cell death in the developing cerebral cortex: role of brain-derived neurotrophic factor and its signaling pathways. J. Neurosci. Res. 68, 213–225. 10.1002/jnr.10208 11948666

[B29] Cohen G. Collins M. (1970). Alkaloids from catecholamines in adrenal tissue: possible role in alcoholism. Science 167, 1749–1751. 10.1126/science.167.3926.1749 5461272

[B507] Collins M. A. Ung-Chhun N. Cheng B. Y. Pronger D. (1990). Brain and plasma tetrahydroisoquinolines in rats: effects of chronic ethanol intake and diet. J. Neurochem. 55, 1507–1514. 10.1111/j.1471-4159.1990.tb04932.x 2213007

[B30] Collins M. A. Bigdeli M. G. (1975). Tetrahydroisoquinolines *in vivo*. I. Rat brain formation of salsolinol, a condensation product of dopamine and acetaldehyde, under certain conditions during ethanol intoxication. Life Sci. 16, 585–601. 1168298

[B31] Coonfield D. L. Hill K. G. Kaczmarek H. J. Ferraro F. M. Kiefer S. W. (2002). Low doses of naltrexone reduce palatability and consumption of ethanol in outbred rats. Alcohol 26, 43–47. 10.1016/s0741-8329(01)00180-x 11958946

[B32] Correa M. Arizzi M. N. Betz A. Mingote S. Salamone J. D. (2003). Open field locomotor effects in rats after intraventricular injections of ethanol and the ethanol metabolites acetaldehyde and acetate. Brain Res. Bull. 62, 197–202. 10.1016/j.brainresbull.2003.09.013 14698353

[B33] Correa M. Arizzi-Lafrance M. N. Salamone J. D. (2009). Infusions of acetaldehyde into the arcuate nucleus of the hypothalamus induce motor activity in rats. Life Sci. 84, 321–327. 10.1016/j.lfs.2008.12.013 19146861

[B34] Correa M. Manrique H. M. Font L. Escrig M. A. Aragon C. M. G. (2008). Reduction in the anxiolytic effects of ethanol by centrally formed acetaldehyde: the role of catalase inhibitors and acetaldehyde-sequestering agents. Psychopharmacology 200, 455–464. 10.1007/s00213-008-1219-3 18587667

[B35] Correa M. Miquel M. Sanchis-Segura C. Aragon C. M. G. (1999a). Acute lead acetate adAcute lead acetate administration potentiate-induced locomotor activity in mice: the role of brain catalase. Alcohol. Clin. Exp. Res. 23, 799–805. 10.1097/00000374-199905000-00006 10371398

[B36] Correa M. Miquel M. Sanchis-Segura C. Aragon C. M. G. (1999b). Effects of chronic lead administration on ethanol-induced locomotor and brain catalase activity. Alcohol 19, 43–49. 10.1016/s0741-8329(99)00023-3 10487387

[B37] Correa M. Salamone J. D. Segovia K. N. Pardo M. Longoni R. Spina L.. (2012). Piecing together the puzzle of acetaldehyde as a neuroactive agent. Neurosci. Biobehav. Rev. 36, 404–430. 10.1016/j.neubiorev.2011.07.009 21824493

[B38] Crews F. T. Mdzinarishvili A. Kim D. He J. Nixon K. (2006). Neurogenesis in adolescent brain is potently inhibited by ethanol. Neuroscience 137, 437–445. 10.1016/j.neuroscience.2005.08.090 16289890

[B39] Crews F. T. Sarkar D. K. Qin L. Zou J. Boyadjieva N. Vetreno R. P. (2015). Neuroimmune function and the consequences of alcohol exposure. Alcohol Res. 37, 331–351, 26695754 10.35946/arcr.v37.2.15 PMC4590627

[B40] Crow K. E. Hardman M. J. (1989). “Regulation of rates of ethanol metabolism,” in Regulation, Enzymology, and Metabolites of Ethanol, Human Metabolism of Alcohol, eds Crow K. E. Batt R. D. (Boca Raton, FL: CRC Press), 3–16.

[B41] D’Addario C. Caputi F. F. Rimondini R. Gandolfi O. Del Borrello E. Candeletti S.. (2013). Different alcohol exposures induce selective alterations on the expression of dynorphin and nociceptin systems related genes in rat brain. Addict. Biol. 18, 425–433. 10.1111/j.1369-1600.2011.00326.x 21507157

[B42] D’Addario C. Johansson S. Candeletti S. Romualdi P. Ögren S. O. Terenius L. (2011). Ethanol and acetaldehyde exposure induces specific epigenetic modifications in the prodynorphin gene promoter in a human neuroblastoma cell line. FASEB J. 25, 1069–1075. 10.1096/fj.10-168534 21106935

[B43] D’Addario C. Ming Y. Ogren S. O. Terenius L. (2008). The role of acetaldehyde in mediating effects of alcohol on expression of endogenous opioid system genes in a neuroblastoma cell line. FASEB J. 22, 662–670. 10.1096/fj.07-8346com 17934066

[B44] Dar N. J. Hamid A. Ahmad M. (2015). Pharmacologic overview of Withania somnifera, the Indian Ginseng. Cell. Mol. Life Sci. 72, 4445–4460. 10.1007/s00018-015-2012-1 26306935 PMC11113996

[B45] Davis V. E. Walsh M. J. (1970). Alcohol, amines, and alkaloids: a possible biochemical basis for alcohol addiction. Science 167, 1005–1007. 10.1126/science.167.3920.1005 5460776

[B46] Deehan G. A. Hauser S. R. Wilden J. A. Truitt W. A. Rodd Z. A. (2013). Elucidating the biological basis for the reinforcing actions of alcohol in the mesolimbic dopamine system: the role of active metabolites of alcohol. Front. Behav. Neurosci. 7:104. 10.3389/fnbeh.2013.00104 23986666 PMC3750600

[B47] Deitrich R. (2004). Acetaldehyde: déjà vu du jour. J. Stud. Alcohol. 65, 557–572. 10.15288/jsa.2004.65.557 15536764

[B48] Deitrich R. (2011). Ethanol as a prodrug: brain metabolism of ethanol mediates its reinforcing effects-a commentary. Alcohol. Clin. Exp. Res. 35, 581–583. 10.1111/j.1530-0277.2011.01454.x 21352247

[B49] Deitrich R. A. Bludeau P. Roper M. Schmuck J. (1978). Induction of aldehyde dehydrogenases. Biochem. Pharmacol. 27, 2343–2347. 10.1016/0006-2952(78)90142-9 103552

[B50] Dellarco V. L. (1988). Mutagenicity assessment of acetaldehyde. Mutat. Res. 195, 1–20. 10.1016/0165-1110(88)90013-9 3122032

[B51] Deng X. S. Deitrich R. A. (2008). Putative role of brain acetaldehyde in ethanol addiction. Clinurr. Drug Abuse Rev. 1, 3–8. 10.2174/1874473710801010003 19122804 PMC2613359

[B52] Diana M. Peana A. T. Sirca D. Lintas A. Melis M. Enrico P. (2008). Crucial role of acetaldehyde in alcohol activation of the mesolimbic dopamine system. Ann. N Y Acad. Sci. 1139, 307–317. 10.1196/annals.1432.009 18991876

[B53] Donohue T. M. Jr. Thomes P. G. (2014). Ethanol-induced oxidant stress modulates hepatic autophagy and proteasome activity. Redox Biol. 3, 29–39. 10.1016/j.redox.2014.10.006 25462063 PMC4297932

[B54] Duncan C. Deitrich R. A. (1980). A critical evaluation of tetrahydroisoquinoline induced ethanol preference in rats. Pharmacol. Biochem. Behav. 13, 265–281. 10.1016/0091-3057(80)90083-0 7413698

[B55] Enrico P. Sirca D. Mereu M. Peana A. T. Lintas A. Golosio A.. (2009). Acetaldehyde sequestering prevents ethanol-induced stimulation of mesolimbic dopamine transmission. Drug Alcohol Depend. 100, 265–271. 10.1016/j.drugalcdep.2008.10.010 19070441

[B56] Erdozain A. M. Callado L. F. (2014). Neurobiological alterations in alcohol addiction: a review. Adicciones 26, 360–370. 25578004

[B57] Erdozain A. M. Morentin B. Bedford L. King E. Tooth D. Brewer C.. (2014). Alcohol-related brain damage in humans. PLoS One 9:e93586. 10.1093/med/9780199696758.003.0051 24699688 PMC3974765

[B58] Eriksson C. J. P. (2001). The role of acetaldehyde in the actions of alcohol (update 2000). Alcohol. Clin. Exp. Res. 25, 15S–32S. 10.1111/j.1530-0277.2001.tb02369.x 11391045

[B59] Eriksson C. J. Fukunaga T. (1993). Human blood acetaldehyde (update 1992). Alcohol Alcohol. 2, 9–25. 7748353

[B60] Eriksson C. J. Sippel H. W. (1977). The distribution and metabolism of acetaldehyde in rats during ethanol oxidation-I. The distribution of acetaldehyde in liver, brain, blood and breath. Biochem. Pharmacol. 26, 241–247. 10.1016/0006-2952(77)90310-0 843394

[B61] Escarabajal M. D. Aragon C. M. G. (2002). Concurrent administration of diethyldithiocarbamate and 4-methylpyrazole enhances ethanol-induced locomotor activity: the role of brain ALDH. Psychopharmacology 160, 339–343. 10.1007/s00213-001-0991-0 11919660

[B62] Fernandes P. A. Ribeiro A. M. Pereira R. F. Marra H. L. Pittella J. E. (2002). Chronic ethanol intake and ageing effects on cortical and basal forebrain cholinergic parameters: morphometric and biochemical studies. Addict. Biol. 7, 29–36. 10.1080/135562101200100571 11900620

[B63] Fertel R. H. Greenwald J. E. Schwarz R. Wong L. Bianchine J. (1980). Opiate receptor binding and analgesic effects of the tetrahydroisoquinolines salsolinol and tetrahydropapaveroline. Res. Commun. Chem. Pathol. Pharmacol. 27, 3–16. 6244607

[B64] Flatscher-Bader T. Van der Brug M. Hwang J. W. Gochee P. A. Matsumoto I. Niwa S.. (2005). Alcohol-responsive genes in the frontal cortex and nucleus accumbens of human alcoholics. J. Neurochem. 93, 359–370. 10.1111/j.1471-4159.2004.03021.x 15816859

[B65] Flatscher-Bader T. Wilce P. A. (2008). Impact of alcohol abuse on protein expression of midkine and excitatory amino acid transporter 1 in the human prefrontal cortex. Alcohol. Clin. Exp. Res. 32, 1849–1858. 10.1111/j.1530-0277.2008.00754.x 18657127

[B66] Flentke G. R. Garic A. Hernandez M. Smith S. M. (2014). CaMKII represses transcriptionally active β-catenin to mediate acute ethanol neurodegeneration and can phosphorylate β-catenin. J. Neurochem. 128, 523–535. 10.1111/jnc.12464 24117889 PMC3946158

[B67] Foddai M. Dosia G. Spiga S. Diana M. (2004). Acetaldehyde increases dopaminergic neuronal activity in the VTA. Neuropsychopharmacology 29, 530–536. 10.1038/sj.npp.1300326 14973432

[B68] Fois G. R. Diana M. (2016). Opioid antagonists block acetaldehyde-induced increments in dopamine neurons activity. Drug Alcohol Depend. 158, 172–176. 10.1016/j.drugalcdep.2015.11.013 26652895

[B69] Font L. Aragon C. M. G. Miquel M. (2006). Voluntary ethanol consumption decreases after the inactivation of central acetaldehyde by D-penicillamine. Behav. Brain Res. 171, 78–86. 10.1016/j.bbr.2006.03.020 16621047

[B70] Font L. Luján M. Á. Pastor R. (2013). Involvement of the endogenous opioid system in the psychopharmacological actions of ethanol: the role of acetaldehyde. Front. Behav. Neurosci. 7:93. 10.3389/fnbeh.2013.00093 23914161 PMC3728478

[B71] Font L. Miquel M. Aragon C. M. G. (2008). Involvement of brain catalase activity in the acquisition of ethanol-induced conditioned place preference. Physiol. Behav. 93, 733–741. 10.1016/j.physbeh.2007.11.026 18155096

[B72] Galter D. Carmine A. Buervenich S. Duester G. Olson L. (2003). Distribution of classI, III and IV alcohol dehydrogenase mRNAs in the adult rat, mouse and human brain. Eur. J. Biochem. 270, 1316–1326. 10.1046/j.1432-1033.2003.03502.x 12631290

[B73] Gessa G. L. Muntoni F. Collu M. Vargiu L. Mereu G. (1985). Low doses of ethanol activate dopaminergic neurons in the ventral tegmental area. Brain Res. 348, 201–203. 10.1016/0006-8993(85)90381-6 2998561

[B74] Ghozland S. Chu K. Kieffer B. L. Roberts A. J. (2005). Lack of stimulant and anxiolytic-like effects of ethanol and accelerated development of ethanol dependence in mu-opioid receptor knockout mice. Neuropharmacology 49, 493–501. 10.1016/j.neuropharm.2005.04.006 15961126

[B75] Gill K. Liu Y. Deitrich R. A. (1996). Voluntary alcohol consumption in BXD recombinant inbred mice: relationship to alcohol metabolism. Alcohol. Clin. Exp. Res. 20, 185–190. 10.1111/j.1530-0277.1996.tb01063.x 8651451

[B76] Grant K. A. Samson H. H. (1986). The induction of oral ethanol self-administration by contingent ethanol delivery. Drug Alcohol Depend. 16, 361–368. 10.1016/0376-8716(86)90069-4 3698814

[B77] Guan Y. Z. Ye J.-H. (2010). Ethanol blocks long-term potentiation of GABAergic synapses in the ventral tegmental area involving μ-opioid receptors. Neuropsychopharmacology 35, 1841–1849. 10.1038/npp.2010.51 20393452 PMC2904870

[B78] Haber H. Dumaual N. Bare D. J. Melzig M. F. McBride W. F. Lumeng L.. (1999). The quantitative determination of R- and S-salsolinol in the striatum and adrenal gland of rats selectively bred for disparate alcohol drinking. Addict. Biol. 4, 181–189. 10.1080/13556219971687 20575784

[B79] Hamby-Mason R. Chen J. J. Schenker S. Perez A. Henderson G. I. (1997). Catalase mediates acetaldehyde formation from ethanol in fetal and neonatal rat brain. Alcohol. Clin. Exp. Res. 21, 1063–1072. 10.1097/00000374-199709000-00020 9309319

[B80] Handler J. A. Thurman R. G. (1988a). Catalase-dependent ethanol oxidation in perfused rat liver Requirement for fatty-acid-stimulated H_2_O_2_ production by peroxisomes. Eur. J. Biochem. 176, 477–484. 10.1111/j.1432-1033.1988.tb14305.x 3416882

[B81] Handler J. A. Thurman R. G. (1988b). Hepatic ethanol metabolism is mediated predominantly by catalase-H_2_O_2_ in the fasted state. FEBS Lett. 238, 139–141. 10.1016/0014-5793(88)80243-6 3169246

[B82] Haorah J. Knipe B. Leibhart J. Ghorpade A. Persidsky Y. (2005). Alcohol-induced oxidative stress in brain endothelial cells causes blood-brain barrier dysfunction. J. Leukoc. Biol. 78, 1223–1232. 10.1189/jlb.0605340 16204625

[B83] Haorah J. Ramirez S. H. Floreani N. Gorantla S. Morsey B. Persidsky Y. (2008). Mechanism of alcohol-induced oxidative stress and neuronal injury. Free Radic. Biol. Med. 45, 1542–1550. 10.1016/j.freeradbiomed.2008.08.030 18845238 PMC2605399

[B84] Haorah J. Ramirez S. H. Schall K. Smith D. Pandya R. Persidsky Y. (2007). Oxidative stress activates protein tyrosine kinase and matrix metalloproteinases leading to blood-brain barrier dysfunction. J. Neurochem. 101, 566–576. 10.1111/j.1471-4159.2006.04393.x 17250680

[B85] Harris R. A. Bajo M. Bell R. L. Blednov Y. A. Varodayan F. P. Truitt J. M.. (2017). Genetic and pharmacologic manipulation of TLR4 has minimal impact on ethanol consumption in rodents. J. Neurosci. 37, 1139–1155. 10.1523/jneurosci.2002-16.2016 27986929 PMC5296793

[B86] Haseba T. Ohno Y. (1997). Assay methods of alcohol metabolizing enzymes for the research on enzymatic mechanism of alcohol metabolism. Nippon Rinnshou. 55, 665–673. 10.3390/ijerph7031076 9078807

[B87] Haseba T. Ohno Y. (2010). A new view of alcohol metabolism and alcoholism—role of the high-K_m_ Class III alcohol dehydrogenase (ADH3). Int. J. Environ. Res. Public Health 7, 1076–1092. 10.3390/ijerph7031076 20617019 PMC2872310

[B88] Haseba T. Tomita Y. Kurosu M. Ohno Y. (2003). Dose and time changes in liver alcohol dehydrogenase (ADH) activity during acute alcohol intoxication involve not only class I but also class III ADH and govern elimination rate of blood ethanol. Legal Med. 5, 202–211. 10.1016/s1344-6223(03)00080-4 14602163

[B89] He D. Chen H. Muramatsu H. Lasek A. W. (2015). Ethanol activates midkine and anaplastic lymphoma kinase signaling in neuroblastoma cells and in the brain. J. Neurochem. 135, 508–521. 10.1111/jnc.13252 26206265 PMC4618084

[B90] Heap L. Ward R. J. Abiaka C. Dexter D. Lawlor M. Pratt O.. (1995). The influence of brain acetaldehyde on oxidative status, dopamine metabolism and visual discrimination task. Biochem. Pharmacol. 50, 263–270. 10.1016/0006-2952(94)00539-x 7632171

[B91] Hellmann J. Rommelspacher H. Wernicke C. (2009). Long term ethanol exposure impairs neuronal differentiation of human neuroblastoma cells involving neurotrophin-mediated intracellular signaling and in particular protein kinase C. Alcohol. Clin. Exp. Res. 33, 538–550. 10.1111/j.1530-0277.2008.00867.x 19120063

[B92] Hempel J. Bühler R. Kaiser R. Holmquist B. de Zalenski C. von Wartburg J. P.. (1984). Human liver alcohol dehydrogenase 1. The primary structure of the β 1 β 1 isoenzyme. Eur. J. Biochem. 145, 437–445. 6391920 10.1111/j.1432-1033.1984.tb08573.x

[B93] Hernández J. A. López-Sánchez R. C. Rendón-Ramírez A. (2016). Lipids and oxidative stress associated with ethanol-induced neurological damage. Oxid. Med. Cell. Longev. 2016:1543809. 10.1155/2016/1543809 26949445 PMC4753689

[B94] Herradón G. Pérez-García C. (2014). Targeting midkine and pleiotrophin signalling pathways in addiction and neurodegenerative disorders: recent progress and perspectives. Br. J. Pharmacol. 171, 837–848. 10.1111/bph.12312 23889475 PMC3925022

[B95] Hipólito L. Martí-Prats L. Sánchez-Catalán M. J. Polache A. Granero L. (2011). Inductio of conditioned place preference and dopamine release by salsolinol in posterior VTA of rats: involvement of μ-opioid receptors. Neurochem. Int. 59, 559–562. 10.1016/j.neuint.2011.04.014 21693150

[B97] Hipólito L. Sánchez-Catalán M. J. Granero L. Polache A. (2009). Local salsolinol modulates dopamine extracellular levels from rat nucleus accumbens: shell/core differences. Neurochem. Int. 55, 187–192. 10.1016/j.neuint.2009.02.014 19524107

[B98] Hipólito L. Sánchez-Catalán M. J. Martí-Prats L. Granero L. Polache A. (2012). Revisiting the controversial role of salsolinol in the neurobiological effects ofethanol: old and new vistas. Neurosci. Biobehav. Rev. 36, 362–378. 10.1016/j.neubiorev.2011.07.007 21802444

[B96] Hipólito L. Sanchez M. J. Polache A. Granero L. (2007). Brain metabolism of ethanol and alcoholism: an update. Curr. Drug Metab. 8, 716–727. 10.2174/138920007782109797 17979660

[B99] Hipólito L. Sánchez-Catalán M. J. Zanolini I. Polache A. Granero L. (2008). Shell/core differences in mu- and delta-opioid receptor modulation of dopamine efflux in nucleus accumbens. Neuropharmacology 55, 183–189. 10.1016/j.neuropharm.2008.05.012 18582908

[B100] Hipólito L. Sánchez-Catalán M. J. Zornoza T. Polache A. Granero L. (2010). Locomotor stimulant effects of acute and repeated intrategmental injections of salsolinol in rats: role of mu-opioid receptors. Psychopharmacology 209, 1–11. 10.1007/s00213-009-1751-9 20084370

[B101] Homicsko K. G. Kertesz I. Radnai B. Toth B. E. Toth G. Fulop F.. (2003). Binding site of salsolinol: its properties in different regions of the brain and the pituitary gland of the rat. Neurochem. Int. 42, 19–26. 10.1016/s0197-0186(02)00063-3 12441164

[B102] Hoover D. J. Brien J. F. (1981). Acetaldehyde concentration in rat blood and brain during the calcium carbimide-ethanol interaction. Can. J. Physiol. Pharmacol. 59, 65–70. 10.1139/y81-011 7214210

[B103] Ibba F. Vinci S. Spiga S. Peana A. T. Assaretti A. R. Spina L.. (2009). Ethanol-induced extracellular signal regulated kinase: role of dopamine D1 receptors. Alcohol Clin. Exp. Res. 33, 858–867. 10.1111/j.1530-0277.2009.00907.x 19320634

[B104] Israel Y. Ezquer F. Quintanilla M. E. Morales P. Ezquer M. Herrera-Marschitz M. (2017). Intracerebral stem cell administration inhibits relapse-like alcohol drinking in rats. Alcohol Alcohol 52, 1–4. 10.1093/alcalc/agw068 27651282

[B105] Israel Y. Quintanilla M. E. Karahanian E. Rivera-Meza M. Herrera-Marschitz M. (2015). The “first hit” toward alcohol reinforcement: role of ethanol metabolites. Alcohol. Clin. Exp. Res. 39, 776–786. 10.1111/acer.12709 25828063

[B106] Jalabert M. Bourdy R. Courtin J. Veinante P. Manzoni O. J. Barrot M.. (2011). Neuronal circuits underlying acute morphine action on dopamine neurons. Proc. Natl. Acad. Sci. U S A 108, 16446–16450. 10.1073/pnas.1105418108 21930931 PMC3182694

[B107] Jamal M. Ameno K. Ameno S. Okada N. Ijiri I. (2003a). Effect of different doses of cyanamide on striatal salsolinol formation after ethanol treatment. Legal Med. 5, S79–S82. 10.1016/s1344-6223(02)00083-4 12935558

[B108] Jamal M. Ameno K. Ameno S. Okada N. Ijiri I. (2003b). *In vivo* study of salsolinol produced by a high concentration of acetaldehyde in the striatum and nucleus accumbens of free-moving rats. Alcohol. Clin. Exp. Res. 27, 79S–84S. 10.1097/01.ALC.0000078617.33026.AD 12960514

[B110] Jamal M. Ameno K. Kubota T. Ameno S. Zhang X. Kumihashi M.. (2003c). *In vivo* formation of salsolinol induced by high acetaldehyde concentration in rat striatum employing microdyalisis. Alcohol Alcohol. 38, 197–201. 10.1093/alcalc/agg056 12711651

[B109] Jamal M. Ameno K. Ikuo U. Kumihashi M. Wang W. Ijiri I. (2007). Ethanol and acetaldehyde: *in vivo* quantitation and effects on cholinergic function in rat brain. Novartis Found. Symp. 285, 137–141. 17590992 10.1002/9780470511848.ch10

[B111] Johnson S. W. North R. A. (1992). Opioids excite dopamine neurons by hyperpolarization of local interneurons. J. Neurosci. 12, 483–488. 1346804 10.1523/JNEUROSCI.12-02-00483.1992 PMC6575608

[B112] Karahanian E. Quintanilla M. E. Tampier L. Rivera-Meza M. Bustamante D. Gonzalez-Lira V.. (2011). Ethanol as a prodrug: brain metabolism of ethanol mediates its reinforcing effects. Alcohol. Clin. Exp. Res. 35, 606–612. 10.1111/j.1530-0277.2011.01454.x 21332529 PMC3142559

[B113] Karahanian E. Rivera-Meza M. Tampier L. Quintanilla M. E. Herrera-Marschitz M. Israel Y. (2015). Long-term inhibition of ethanol intake by the administration of an aldehyde dehydrogenase-2 (ALDH2)-coding lentiviral vector into the ventral tegmental area of rats. Addict. Biol. 20, 336–344. 10.1111/adb.12130 24571199

[B114] Kiessling K. H. (1962). The occurrence of acetaldehyde in various parts of rat brain after alcohol injection and its effect on pyruvate oxidation. Exp. Cell. Res. 27, 367–368. 10.1016/0014-4827(62)90248-3 14455856

[B115] Kim J. S. Shukla S. D. (2006). Acute *in vivo* effect of ethanol (binge drinking) on histone H3 modifications in rat tissues. Alcohol Alcohol. 41, 126–132. 10.1093/alcalc/agh248 16314425

[B116] Koechling U. M. Amit Z. (1994). Effects of 3-amino-1,2,4-triazole on brain catalase in the mediation of ethanol consumption in mice. Alcohol 11, 235–239. 10.1016/0741-8329(94)90036-1 8060524

[B117] Koob G. F. Le Moal M. (2006). Neurobiology of Addiction. London: Academic Press.

[B118] Kurnik M. Gil K. Gajda M. Thor P. Bugajski A. (2015). Neuropathic alterations of the myenteric plexus neurons following subacute intraperitoneal administration of salsolinol. Folia Histochem. Cytobiol. 53, 49–61. 10.5603/fhc.a2015.0010 25815627

[B119] Lachenmeier D. W. Salaspuro M. (2017). ALDH2-deficiency as genetic epidemiologic and biochemical model for the carcinogenicity of acetaldehyde. Regul. Toxicol. Pharmacol. 86, 128–136. 10.1016/j.yrtph.2017.02.024 28257851

[B120] Lasek A. W. Lim J. Kliethermes C. L. Berger K. H. Joslyn G. Brush G.. (2011). An evolutionary conserved role for anaplastic lymphoma kinase in behavioral responses to ethanol. PLoS One 6:e22636. 10.1371/journal.pone.0022636 21799923 PMC3142173

[B121] Ledesma J. C. Aragon C. M. G. (2012). Acquisition and reconditioning of ethanol-induced conditioned place preference in mice is blocked by the H_2_O_2_ scavenger alpha lipoic acid. Psychopharmacology 226, 673–685. 10.1007/s00213-012-2831-9 22885873

[B122] Ledesma J. C. Font L. Aragon C. M. G. (2012). The H_2_O_2_ scavenger ebselen decreases ethanol-induced locomotor stimulation in mice. Drug Alcohol Depend. 124, 42–49. 10.1016/j.drugalcdep.2011.12.003 22261181

[B123] Ledesma J. C. Font L. Baliño P. Aragon C. M. (2013). Modulation of ethanol-induced conditioned place preference in mice by 3-amino-1,2,4-triazole and D-penicillamine depends on ethanol dose and number of conditioning trials. Psychopharmacology 230, 557–568. 10.1007/s00213-013-3177-7 23832421

[B124] Lee H. J. Oh S. H. Jang H. W. Kwon J. H. Lee K. J. Kim C. H.. (2016). Long-term effects of bone marrow-derived mesenchymal stem cells in dextran sulfate sodium-induced murine chronic colitis. Gut Liver 10, 412–419. 10.5009/gnl15229 27114436 PMC4849695

[B125] Lee J. Ramchandani V. A. Hamazaki K. Engleman E. A. McBride W. J. Li T. K.. (2010). A critical evaluation of influence of ethanol and diet on salsolinol enantiomers in humans and rats. Alcohol. Clin. Exp. Res. 34, 242–250. 10.1111/j.1530-0277.2009.01087.x 19951298 PMC2858379

[B126] Lieber C. S. (2004). Alcoholic fatty liver: its pathogenesis and mechanism of progression to inflammation and fibrosis. Alcohol 34, 9–19. 10.1016/j.alcohol.2004.07.008 15670660

[B127] Lieber C. S. DeCarli L. M. (1972). The role of the hepatic microsomal ethanol oxidizing system (MEOS) for ethanol metabolism *in vivo*. J. Pharmacol. Exp. Ther. 181, 279–287. 4402282

[B128] Lucchi L. Bosio A. Spano P. F. Trabucchi M. (1982). Action of ethanol and salsolinol on opiate receptor function. Brain Res. 232, 506–510. 10.1016/0006-8993(82)90297-9 6322918

[B129] Mao J. Ma H. Xu Y. Su Y. Zhu H. Wang R.. (2013). Increased levels of monoamine-derived potential neurotoxins in fetal rat brain exposed to ethanol. Neurochem. Res. 38, 356–363. 10.1007/s11064-012-0926-7 23184185

[B130] March S. M. Abate P. Spear N. E. Molina J. C. (2013). The role of acetaldehyde in ethanol reinforcement assessed by pavlovian conditioning in newborn rats. Psychopharmacology 226, 491–499. 10.1007/s00213-012-2920-9 23196716

[B131] Martí-Prats L. Orrico A. Polache A. Granero L. (2015). Dual motor responses elicited by ethanol in the posterior VTA: consequences of the blockade of μ-opioid receptors. J. Psychopharmacol. 29, 1029–1034. 10.1177/0269881115598337 26216379

[B132] Martí-Prats L. Sánchez-Catalán M. J. Orrico A. Zornoza T. Polache A. Granero L. (2013). Opposite motor responses elicited by ethanol in the posterior VTA: the role of acetaldehyde and the non-metabolized fraction of ethanol. Neuropharmacology 72, 204–214. 10.1016/j.neuropharm.2013.04.047 23643753

[B133] Maruyama W. Nakahara D. Ota M. Takahashi T. Takahashi A. Nagatsu T.. (1992). N-methylation of dopamine-derived 6,7-dihydroxy-1,2,3,4-tetrahydroisoquinoline, (R)-salsolinol, in rat brains: *in vivo* microdialysis study. J. Neurochem. 59, 395–400. 10.1111/j.1471-4159.1992.tb09384.x 1629715

[B134] Matsubara K. Fukushima S. Fukui Y. (1987). A systematic regional study of brain salsolinol levels during and immediately following chronic ethanol ingestion in rats. Brain Res. 413, 336–343. 10.1016/0006-8993(87)91025-0 3607483

[B135] Matsuzawa S. Suzuki T. Misawa M. (2000). Involvement of μ-opioid receptor in the salsolinol-associated place preference in rats exposed to conditioned fear stress. Alcohol. Clin. Exp. Res. 24, 366–372. 10.1111/j.1530-0277.2000.tb04624.x 10776678

[B136] McBride W. J. Li T. K. Deitrich R. A. Zimatkin S. Smith B. R. Rodd-Henricks Z. A. (2002). Involvement of acetaldehyde in alcohol addiction. Alcohol. Clin. Exp. Res. 26, 114–119. 10.1111/j.1530-0277.2002.tb02439.x 11821661

[B137] Melchior C. L. Myers R. D. (1977). Preference for alcohol evoked by tetrahydropapaveroline (THP) chronically infused in the cerebral ventricle of the rat. Pharmacol. Biochem. Behav. 7, 19–35. 10.1016/0091-3057(77)90006-5 561961

[B138] Melis M. Carboni E. Caboni P. Acquas E. (2015). Key role of salsolinol in ethanol actions on dopamine neuronal activity of the posterior ventral tegmental area. Addict. Biol. 20, 182–193. 10.1111/adb.12097 24103023

[B139] Melis M. Enrico P. Peana A. T. Diana M. (2007). Acetaldehyde mediates alcohol activation of the mesolimbic dopamine system. Eur. J. Neurosci. 26, 2824–2833. 10.1111/j.1460-9568.2007.05887.x 18001279

[B140] Mereu G. Gessa G. L. (1985). Low doses of ethanol inhibit the firing of neurons in the substantia nigra, pars reticulata: a GABAergic effect? Brain Res. 360, 325–330. 10.1016/0006-8993(85)91249-1 3000533

[B141] Montesinos J. Alfonso-Loeches S. Guerri C. (2016). Impact of the innate immune response in the actions of ethanol on the central nervous system. Alcohol. Clin. Exp. Res. 40, 2260–2270. 10.1111/acer.13208 27650785

[B142] Mooney S. M. Miller M. W. (2011). Role of neurotrophins on postnatal neurogenesis in the thalamus: prenatal exposure to ethanol. Neuroscience 179, 256–266. 10.1016/j.neuroscience.2011.01.046 21277941 PMC3059403

[B143] Moore D. B. Madorsky I. Paiva M. Barrow Heaton M. (2004). Ethanol exposure alters neurotrophin receptor expression in the rat central nervous system: effects of neonatal exposure. J. Neurobiol. 60, 114–126. 10.1002/neu.20010 15188277

[B144] Morikawa H. Morrisett R. A. (2010). Ethanol action on dopaminergic neurons in the ventral tegmental area: interaction with intrinsic ion channels and neurotransmitter inputs. Int. Rev. Neurobiol. 91, 235–288. 10.1016/S0074-7742(10)91008-8 20813245 PMC2936723

[B145] Moulder K. L. Fu T. Melbostad H. Cormier R. J. Isenberg K. E. Zorumski C. F.. (2002). Ethanol-induced death of postnatal hippocampal neurons. Neurobiol. Dis. 10, 396–409. 10.1006/nbdi.2002.0523 12270700

[B146] Mulligan M. K. Ponomarev I. Hitzemann R. J. Belknap J. K. Tabakoff B. Harris R. A.. (2006). Toward understanding the genetics of alcohol drinking through transcriptome meta-analysis. Proc. Natl. Acad. Sci. U S A 103, 6368–6373. 10.1073/pnas.0510188103 16618939 PMC1458884

[B147] Myers R. D. Melchior C. L. (1977). Differential actions on voluntary alcohol intake of tetrahydroisoquinolines or a β-carboline infused chronically in the ventricle of the rat. Pharmacol. Biochem. Behav. 7, 381–392. 10.1016/0091-3057(77)90235-0 563084

[B148] Myers R. D. Robinson D. E. (1999). Tetrahydropapaveroline injected in the ventral tegmental area shifts dopamine efflux differentially in the shell and core of nucleus accumbens in high-ethanol-preferring (HEP) rats. Alcohol 18, 83–90. 10.1016/s0741-8329(99)00008-7 10386670

[B149] Myers W. D. Mackenzie L. Ng K. T. Singer G. Smythe G. A. Duncan M. W. (1985). Salsolinol and dopamine in rat medial basal hypothalamus after chronic ethanol exposure. Life Sci. 36, 309–314. 10.1016/0024-3205(85)90115-8 4038420

[B150] Myers W. D. Ng K. T. Singer G. (1982). Intravenous self-administration of acetaldehyde in the rat as a function of schedule, food deprivation and photoperiod. Pharmacol. Biochem. Behav. 17, 807–811. 10.1016/0091-3057(82)90364-1 7178188

[B151] Nagasawa H. T. Elberling J. A. DeMaster E. G. (1980). Structural requirements for the sequestration of metabolically generated acetaldehyde. J. Med. Chem. 23, 140–143. 10.1002/chin.198026137 7359527

[B152] Nakahara D. Maruyama W. Hashiguti H. Naoi M. (1994). Characterization of the *in vivo* action of *(R)*-salsolinol, an endogenous metabolite of alcohol, on serotonin and dopamine metabolism: a microdialysis study. Brain. Res. 644, 226–232. 10.1016/0006-8993(94)91684-5 8050034

[B153] Nixon K. Crews F. T. (2002). Binge ethanol exposure decreases neurogenesis in adult rat hippocampus. J. Neurochem. 83, 1087–1093. 10.1046/j.1471-4159.2002.01214.x 12437579

[B154] O’Neill P. J. Rahwan R. G. (1977). Absence of formation of brain salsolinol in ethanoldependent mice. J. Pharmacol. Exp. Ther. 200, 306–313. 557108

[B155] Okamoto T. Harnett M. T. Morikawa H. (2006). Hyperpolarization-activated cation current (ih) is an ethanol target in midbrain dopamine neurons of mice. J. Neurophysiol. 95, 619–626. 10.1152/jn.00682.2005 16148268 PMC1454360

[B156] Orrico A. Hipólito L. Sánchez-Catalán M. J. Martí-Prats L. Zornoza T. Granero L.. (2013). Efficacy of D-penicillamine, a sequestering acetaldehyde agent, in the prevention of alcohol relapse-like drinking in rats. Psychopharmacology 228, 563–575. 10.1007/s00213-013-3065-1 23515584

[B157] Orrico A. Martí-Prats L. Cano-Cebrián M. J. Granero L. Polache A. Zornoza T. (2014). Improved effect of the combination naltrexone/D-penicillamine in the prevention of alcohol relapse-like drinking in rats. J. Psychopharmacol. 28, 76–81. 10.1177/0269881113515063 24306132

[B158] Oshino N. Oshino R. Chance B. (1973). The characteristics of the “peroxidatic” reaction of catalase in ethanol oxidation. Biochem. J. 131, 555–563. 10.1042/bj1310555 4720713 PMC1177502

[B159] Oswald L. M. Wand G. S. (2004). Opioids and alcoholism. Physiol. Behav. 81, 339–358. 10.1016/j.physbeh.2004.02.008 15159175

[B160] Pandey S. C. Zhang H. Ugale R. Prakash A. Xu T. Misra K. (2008). Effector immediate-early gene arc in the amygdala plays a critical role in alcoholism. J. Neurosci. 28, 2589–2600. 10.1523/jneurosci.4752-07.2008 18322102 PMC6671198

[B161] Pascual M. Baliño P. Aragon C. M. G. Guerri C. (2015). Cytokines and chemokines as biomarkers of ethanol-induced neuroinflammation and anxiety-related behavior: role of TLR4 and TLR2. Neuropharmacology 89, 352–359. 10.1016/j.neuropharm.2014.10.014 25446779

[B162] Pastor R. Aragon C. M. G. (2008). Ethanol injected into the hypothalamic arcuate nucleus induces behavioral stimulation in rats: an effect prevented by catalase inhibition and naltrexone. Behav. Pharmacol. 19, 698–705. 10.1097/fbp.0b013e328315ecd7 18797246

[B163] Pautassi R. M. Nizhnikov M. E. Fabio M. C. Spear N. E. (2011). An acetaldehyde-sequestering agent inhibits appetitive reinforcement and behavioral stimulation induced by ethanol in preweanling rats. Pharmacol. Biochem. Behav. 97, 462–469. 10.1016/j.pbb.2010.10.005 20951160 PMC3214973

[B164] Peana A. T. Acquas E. (2013). Behavioral and biochemical evidence of the role of acetaldehyde in the motivational effects of ethanol. Front. Behav. Neurosci. 7:86. 10.3389/fnbeh.2013.00086 23874276 PMC3710953

[B165] Peana A. T. Assaretti A. R. Muggironi G. Enrico P. Diana M. (2009). Reduction of ethanol-derived acetaldehyde induced motivational properties by L-cysteine. Alcohol. Clin. Exp. Res. 33, 43–48. 10.1111/j.1530-0277.2008.00809.x 18945224

[B166] Peana A. T. Enrico P. Assaretti A. R. Pulighe E. Muggironi G. Nieddu M.. (2008). Key role of ethanol-derived acetaldehyde in the motivational properties induced by intragastric ethanol: a conditioned place preference study in the rat. Alcohol. Clin. Exp. Res. 32, 249–258. 10.1111/j.1530-0277.2007.00574.x 18162073

[B167] Peana A. T. Giugliano V. Rosas M. Sabariego M. Acquas E. (2013a). Effects of L-cysteine on reinstatement of ethanol-seeking behavior and on reinstatement-elicited extracellular signal-regulated kinase phosphorylation in the rat nucleus accumbens shell. Alcohol. Clin. Exp. Res. 37, E329–E337. 10.1111/j.1530-0277.2012.01877.x 22823513

[B170] Peana A. T. Muggironi G. Fois G. Diana M. (2013b). Alpha- lipoic acid reduces ethanol self- administration in rats. Alcohol. Clin. Exp. Res. 37, 1816–1822. 10.1111/acer.12169 23802909

[B168] Peana A. T. Muggironi G. Calvisi G. Enrico P. Mereu M. Nieddu M.. (2010a). L-cysteine reduces oral ethanol self-administration and reinstatement of ethanol-drinking behavior in rats. Pharmacol. Biochem. Behav. 94, 431–437. 10.1016/j.pbb.2009.10.005 19879891

[B169] Peana A. T. Muggironi G. Diana M. (2010b). Acetaldehyde motivational effects; a study on oral self-administration behavior. Front. Psychiatry 1:23 10.3389/fpsyt.2010.00023 21423434 PMC3059631

[B171] Peana A. T. Muggironi G. Fois G. R. Zinellu M. Sirca D. Diana M. (2012). Effect of (L)-cysteine on acetaldehyde self-administration. Alcohol 46, 489–497. 10.1016/j.alcohol.2011.10.004 22440691

[B172] Peana A. T. Muggironi G. Fois G. R. Zinellu M. Vinci S. Acquas E. (2011). Effect of opioid receptor blockade on acetaldehyde self-administration and ERK phosphorylation in the rat nucleus accumbens. Alcohol 45, 773–783. 10.1016/j.alcohol.2011.06.003 21803531

[B173] Peana A. T. Muggironi G. Spina L. Rosas M. Kasture S. B. Cotti E.. (2014). Effects of Withania somnifera on oral ethanol self-administration in rats. Behav. Pharmacol. 25, 618–628. 10.1097/fbp.0000000000000078 25115596

[B174] Peana A. T. Porcheddu V. Bennardini F. Carta A. Rosas M. Acquas E. (2015). Role of ethanol-derived acetaldehyde in operant oral self-administration of ethanol in rats. Psychopharmacology 232, 4269–4276. 10.1007/s00213-015-4049-0 26292801

[B175] Peana A. T. Rosas M. Porru S. Acquas E. (2016). From Ethanol to Salsolinol: role of Ethanol metabolites in the effects of Ethanol. J. Exp. Neurosci. 10, 137–146. 10.4137/jen.s25099 27891052 PMC5117487

[B176] Pflaum T. Hausler T. Baumung C. Ackermann S. Kuballa T. Rehm J.. (2016). Carcinogenic compounds in alcoholic beverages: an update. Arch. Toxicol. 90, 2349–2367. 10.1007/s00204-016-1770-3 27353523

[B177] Pikkarainen P. H. Lieber C. S. (1980). Concentration dependency of ethanol elimination rates in baboons: effect of chronic alcohol consumption. Alcohol. Clin. Exp. Res. 4, 40–43. 10.1111/j.1530-0277.1980.tb04789.x 6766681

[B178] Plescia F. Brancato A. Venniro M. Maniaci G. Cannizzaro E. Sutera F. M.. (2015). Acetaldehyde self-administration by a two-bottle choice paradigm: consequences on emotional reactivity, spatial learning, and memory. Alcohol 49, 139–148. 10.1016/j.alcohol.2015.01.002 25636827

[B179] Plotnikov E. Korotkova E. Voronova O. Sazhina N. Petrova E. Artamonov A.. (2016). Comparative investigation of antioxidant activity of human serum blood by amperometric, voltammetric and chemiluminescent methods. Arch. Med. Sci. 12, 1071–1076. 10.5114/aoms.2015.50234 27695499 PMC5016571

[B180] Puig J. G. Fox I. H. (1984). Ethanol-induced activation of adenine nucleotide turnover. Evidence for a role of acetate. J. Clin. Invest. 74, 936–941. 10.1172/jci111512 6470146 PMC425250

[B181] Quertemont E. De Witte P. (2001). Conditioned stimulus preference after acetaldehyde but not ethanol injections. Pharmacol. Biochem. Behav. 68, 449–454. 10.1016/s0091-3057(00)00486-x 11325398

[B182] Quertemont E. Eriksson C. J. Zimatkin S. M. Pronko P. S. Diana M. Pisano M.. (2005). Is ethanol a pro-drug? Acetaldehyde contribution to brain ethanol effects. Alcohol. Clin. Exp. Res. 29, 1514–1521. 10.1097/01.alc.0000175015.51329.45 16156048

[B183] Quertemont E. Tambour S. Bernaerts P. Zimatkin S. M. Tirelli E. (2004). Behavioral characterization of acetaldehyde in C57BL/6J mice: locomotor, hypnotic, anxiolytic, and amnesic effects. Psychopharmacology 177, 84–92. 10.1007/s00213-004-1911-x 15160264

[B184] Quintanilla M. E. Callejas O. Tampier L. (2002). Aversion to acetaldehyde: differences in low-alcohol-drinking (UChA) and high-alcohol-drinking (UChB) rats. Alcohol 26, 69–74. 10.1016/S0741-8329(01)00197-5 12007581

[B186] Quintanilla M. E. Rivera-Meza M. Berrios-Cárcamo P. A. Bustamante D. Buscaglia M. Morales P.. (2014). Salsolinol, free of isosalsolinol, exerts ethanol-like motivational/sensitization effects leading to increases in ethanol intake. Alcohol 48, 551–559. 10.1016/j.alcohol.2014.07.003 25086835

[B185] Quintanilla M. E. Rivera-Meza M. Berríos-Cárcamo P. Cassels B. K. Herrera-Marschitz M. Israel Y. (2016). (*R*)-Salsolinol, a product of ethanol metabolism, stereospecifically induces behavioral sensitization and leads to excessive alcohol intake. Addict. Biol. 21, 1063–1071. 10.1111/adb.12268 26032572

[B187] Reiff T. Huber L. Kramer M. Delattre O. Janoueix-Lerosey I. Rohrer H. (2011). Midkine and Alk signaling in sympathetic neuron proliferation and neuroblastoma predisposition. Development 138, 4699–4708. 10.1242/dev.072157 21989914

[B188] Rodd Z. A. Bell R. L. Zhang Y. Goldstein A. Zaffaroni A. McBride W. J.. (2003). Salsolinol produces reinforcing effects in the nucleus accumbens shell of alcohol-preferring (P) rats. Alcohol. Clin. Exp. Res. 27, 440–449. 10.1097/01.alc.0000056612.89957.b4 12658109

[B189] Rodd Z. A. Bell R. L. Zhang Y. Murphy J. M. Goldstein A. Zaffaroni A.. (2005). Regional heterogeneity for the intracranial self-administration of ethanol and acetaldehyde within the ventral tegmental area of alcohol-preferring (P) rats: involvement of dopamine and serotonin. Neuropsychopharmacology 30, 330–338. 10.1038/sj.npp.1300561 15383830

[B190] Rodd Z. A. Melendez R. I. Bell R. L. Kuc K. A. Zhang Y. Murphy J. M.. (2004). Intracranial self-administration of ethanol within the ventral tegmental area of male wistar rats: evidence for involvement of dopamine neurons. J. Neurosci. 24, 1050–1057. 10.1523/jneurosci.1319-03.2004 14762123 PMC6793578

[B191] Rodd Z. A. Oster S. M. Ding Z. M. Toalston J. E. Deehan G. Bell R. L.. (2008). The reinforcing properties of salsolinol in the ventral tegmental area: evidence for regional heterogeneity and the involvement of serotonin and dopamine. Alcohol. Clin. Exp. Res. 32, 230–239. 10.1111/j.1530-0277.2007.00572.x 18162075

[B192] Rodd-Henricks Z. A. Melendez R. I. Zaffaroni A. Goldstein A. McBride W. J. Li T. K. (2002). The reinforcing effects of acetaldehyde in the posterior ventral tegmental area of alcohol-preferring rats. Pharmacol. Biochem. Behav. 72, 55–64. 10.1016/s0091-3057(01)00733-x 11900769

[B193] Rojkovicova T. Mechref Y. Starkey J. A. Wu G. Bell R. L. McBride W. J. (2008). Quantitative chiral analysis of salsolinol in different brain regions of rats genetically predisposed to alcoholism. J. Chromatogr. B Analyt. Technol. Biomed. Life Sci. 863, 206–214. 10.1016/j.jchromb.2008.01.016 18272438

[B194] Sakai R. Ukai W. Sohma H. Hashimoto E. Yamamoto M. Ikeda H.. (2005). Attenuation of brain derived neurotrophic factor (BDNF) by ethanol and cytoprotective effect of exogenous BDNF against ethanol damage in neuronal cells. J. Neural. Transm. 112, 1005–1013. 10.1007/s00702-004-0246-4 15583957

[B501] Salaspuro M. (2011). Acetaldehyde and gastric cancer. J. Dig. Dis. 12, 51–59. 10.1111/j.1751-2980.2011.00480.x 21401890

[B195] Salaspuro V. Hietala J. Kaihovaara P. Pihlajarinne L. Marvola M. Salaspuro M. (2002). Removal of acetaldehyde from saliva by a slow-release buccal tablet of L-cysteine. Int. J. Cancer 97, 361–364. 10.1002/ijc.1620 11774289

[B196] Samson H. H. Pfeffer A. O. Tolliver G. A. (1988). Oral ethanol self-administration in rats: models of alcohol-seeking behavior. Alcohol. Clin. Exp. Res. 12, 591–598. 10.1111/j.1530-0277.1988.tb00248.x 3067600

[B198] Sánchez-Catalán M. J. Hipólito L. Guerri C. Granero L. Polache A. (2008). Distribution and differential induction of CYP2E1 by ethanol and acetone in the mesocorticolimbic system of rat. Alcohol Alcohol 43, 401–407. 10.1093/alcalc/agn012 18326880

[B197] Sánchez-Catalán M. J. Hipólito L. Zornoza T. Polache A. Granero L. (2009). Motor stimulant effects of ethanol and acetaldehyde injected into the posterior ventral tegmental area of rats: role of opioid receptors. Psychopharmacology 204, 641–653. 10.1007/s00213-009-1495-6 19238363

[B199] Sánchez-Catalán M. J. Kaufling J. Georges F. Veinante P. Barrot M. (2014). The anterio-posterior heterogeneity of the ventral tegmental area. Neuroscience 282, 198–216. 10.1016/j.neuroscience.2014.09.025 25241061

[B200] Sanchis-Segura C. Correa M. Miquel M. Aragon C. M. G. (2005). Catalase inhibition in the Arcuate nucleus blocks ethanol effects on the locomotor activity of rats. Neurosci. Lett. 376, 66–70. 10.1016/j.neulet.2004.11.025 15694276

[B201] Sarkola T. Iles M. R. Kohlenberg-Mueller K. Eriksson C. J. P. (2002). Ethanol, acetaldehyde, acetate and lactate levels after alcohol intake in white men and women: effect of 4-methylpyrazole. Alcohol. Clin. Exp. Res. 26, 239–245. 10.1177/002215540305100606 11964564

[B202] Schad A. Fahimi H. D. Völkl A. Baumgart E. (2003). Expression of catalase mRNA and protein in adult rat brain: detection by nonradioactive *in situ* hybridization with signal amplification by catalyzed reporter deposition (ISH-CARD) and immunohistochemistry (IHC)/immunofluorescence (IF). J. Histochem. Cytochem. 51, 751–760. 10.1177/002215540305100606 12754286

[B504] Seitz H. K. Stickel F. (2007). Molecular mechanisms of alcohol-mediated carcinogenesis. Nat. Rev. Cancer 7, 599–612. 10.1038/nrc2191 17646865

[B203] Seitz H. K. Stickel F. (2010). Acetaldehyde as an underestimated risk factor for cancer development: role of genetics in ethanol metabolism. Genes Nutr. 5, 121–128. 10.1007/s12263-009-0154-1 19847467 PMC2885165

[B506] Sjöquist B. Liljequist S. Engel J. (1982). Increased salsolinol levels in rat striatum and limbic forebrain following chronic ethanol treatment. J. Neurochem. 39, 259–262. 10.1111/j.1471-4159.1982.tb04730.x 7201007

[B204] Serrano E. Pozo O. J. Beltrán J. Hernández F. Font L. Miquel M. (2007). Liquid chromatography/tandem mass spectrometry determination of (*4S,2RS*)-2,5,5-trimethylthiazolidine- 4-carboxylic acid, a stable adduct formed between D-(-)-penicillamine and acetaldehyde (main biological metabolite of ethanol), in plasma, liver and brain rat tissues. Rapid Commun. Mass Spectrom. 21, 1221–1229. 10.1002/rcm.2951 17330215

[B502] Shield K. D. Gmel G. Kehoe-Chan T. Dawson D. A. Grant B. F. Rehm J. (2013). Mortality and potential years of life lost attributable to alcohol consumption by race and sex in the United States in 2005. PLoS One 8: e51923. 10.1371/journal.pone.0051923 23300957 PMC3534703

[B205] Shukla S. D. Aroor A. R. (2006). Epigenetic effects of ethanol on liver and gastrointestinal injury. World J. Gastroenterol. 12, 5265–5271. 10.3748/wjg.v12.i33.5265 16981253 PMC4088190

[B206] Shukla S. D. Lee Y. L. Park P. Aroor A. R. (2007). Acetaldehyde alters MAP kinase signalling and epigenetic histone modifications in hepatocytes. Novartis Found. Symp. 285, 217–224. 10.1002/9780470511848.ch1 17590997

[B207] Sinclair J. D. Lindros K. O. (1981). Suppression of alcohol drinking with brain aldehyde dehydrogenase inhibition. Pharmacol. Biochem. Behav. 14, 377–383. 10.1016/0091-3057(81)90405-6 7232463

[B208] Sippel H. W. (1974). The acetaldehyde content in rat brain during ethanol metabolism. J. Neurochem. 23, 451–452. 10.1111/j.1471-4159.1974.tb04380.x 4417541

[B210] Smith B. R. Aragon C. M. G. Amit Z. (1997). Catalase and the production of brain acetaldehyde: a possible mediator of the psychopharmacological effects of ethanol. Addict. Biol. 2, 277–290. 10.1080/13556219772570 26735784

[B209] Smith B. R. Amit Z. Splawinsky J. (1984). Conditioned place preference induced by intraventricular infusions of acetaldehyde. Alcohol 1, 193–195. 6536284 10.1016/0741-8329(84)90097-1

[B211] Socaransky S. M. Aragon C. M. G. Amit Z. Blander A. (1984). Higher correlation of ethanol consumption with brain than liver aldehyde dehydrogenase in three strains of rats. Psychopharmacology 84, 250–253. 10.1007/bf00427454 6438685

[B212] Soscia S. J. Tong M. Xu X. J. Cohen A. C. Chu J. Wands J. R.. (2006). Chronic gestational exposure to ethanol causes insulin and IGF resistance and impairs acetylcholine homeostasis in the brain. Cell. Mol. Life Sci. 63, 2039–2056. 10.1007/s00018-006-6208-2 16909201 PMC11136445

[B213] Spina L. Longoni R. Rosas M. Collu M. Peana A. T. Espa E.. (2015). *Withania somnifera* Dunal (Indian ginseng) impairs acquisition and expression of ethanol-elicited conditioned place preference and conditioned place aversion. J. Psychopharmacol. 29, 1191–1199. 10.1177/0269881115600132 26349555

[B214] Spina L. Longoni R. Vinci S. Ibba F. Peana A. T. Muggironi G.. (2010). Role of dopamine D_1_ receptors and extracellular signal regulated kinase in the motivational properties of acetaldehyde as assessed by place preference conditioning. Alcohol. Clin. Exp. Res. 34, 607–616. 10.1111/j.1530-0277.2009.01129.x 20102564

[B215] Spivak K. Aragon C. M. G. Amit Z. (1987). Alterations in brain aldehyde dehydrogenase activity modify the locomotor effects produced by ethanol in rats. Alcohol Drug Res. 7, 481–491. 3620014

[B216] Starkey J. A. Mechref Y. Muzikar J. McBride W. J. Novotny M. V. (2006). Determination of salsolinol and related catecholamines through on-line preconcentration and liquid chromatography/atmospheric pressure photoionization mass spectrometry. Anal. Chem. 78, 3342–3347. 10.1021/ac051863j 16689535

[B217] Steffensen S. C. Walton C. H. Hansen D. M. Yorgason J. T. Gallegos R. A. Criado J. R. (2009). Contingent and non-contingent effects of low-dose ethanol on GABA neuron activity in the ventral tegmental area. Pharmacol. Biochem. Behav. 92, 68–75. 10.1016/j.pbb.2008.10.012 18996142 PMC2911963

[B218] Stoica G. E. Kuo A. Powers C. Bowden E. T. Sale E. B. Riegel A. T.. (2002). Midkine binds to anaplastic lymphoma kinase (ALK) and acts as a growth factor for different cell types. J. Biol. Chem. 277, 35990–36008. 10.1074/jbc.m205749200 12122009

[B219] Szabo G. Saha B. (2015). Alcohol’s effect on host defense. Alcohol Res. 37, 159–170. 26695755 10.35946/arcr.v37.2.01 PMC4590613

[B220] Székács D. Bodnár I. Mravec B. Kvetnansky R. Vizi E. S. Nagy G. M.. (2007). The peripheral noradrenergic terminal as possible site of action of salsolinol as prolactoliberin. Neurochem. Int. 50, 427–434. 10.1016/j.neuint.2006.10.001 17141375

[B221] Tabakoff B. Anderson R. A. Ritzmann R. F. (1976). Brain acetaldehyde after ethanol administration. Biochem. Pharmacol. 25, 1305–1309. 10.1016/0006-2952(76)90094-0 938553

[B222] Takagi T. Alderman J. Gellert J. Lieber C. S. (1986). Assessment of the role of non-ADH ethanol oxidation *in vivo* and in hepatocytes from deermice. Biochem. Pharmacol. 35, 3601–3606. 10.1016/0006-2952(86)90632-5 3768042

[B223] Tampier L. Alpers H. S. Davis V. E. (1977). Influence of catecholamine-derived alkaloids and beta-adrenergic blocking agents on stereospecific binding of 3H-naloxone. Res. Commun. Chem. Pathol. Pharmacol. 17, 731–734. 19827

[B224] Tampier L. Mardones J. (1987). Absence of effect of 3-amino-1,2,4-triazole pretreatment on blood ethanol levels after oral administration, in rats. Alcohol 4, 73–74. 10.1016/0741-8329(87)90064-4 3828068

[B225] Tampier L. Quintanilla M. E. Karahanian E. Rivera-Meza M. Herrera-Marschitz M. Israel Y. (2013). The alcohol deprivation effect: marked inhibition by anticatalase gene administration into the ventral tegmental area in rats. Alcohol. Clin. Exp. Res. 37, 1278–1285. 10.1111/acer.12101 23527889

[B226] Tardif R. (2007). The determination of acetaldehyde in exhaled breath. Novartis Found. Symp. 285, 125–133; discussion 133–136, 198–199. 10.1002/9780470511848.ch9 17590991

[B227] Teschke R. Gellert J. (1986). Hepatic microsomal ethanol-oxidizing system (MEOS): metabolic aspects and clinical implications. Alc. Clin. Exp. Res. 10, 20S–32S. 10.1111/j.1530-0277.1986.tb05176.x 3544926

[B228] Tieu K. (2011). A guide to neurotoxic animal models of Parkinson’s disease. Cold Spring Harb. Perspect. Med. 1:a009316. 10.1016/b978-012088382-0/50014-1 22229125 PMC3234449

[B229] Tóth B. E. Bodnár I. Homicskóo K. G. Fülöp F. Fekete M. I. Nagy G. M. (2002). Physiological role of salsolinol: its hypophysiotrophic function in the regulation of pituitary prolactin secretion. Neurotoxicol. Teratol 24, 655–666. 10.1016/S0892-0362(02)00216-7 12200196

[B230] Tuomisto L. Airaksinen M. M. Peura P. Eriksson C. J. (1982). Alcohol drinking in the rat: increases following intracerebroventricular treatment with tetrahydro-β-carbolines. Pharmacol. Biochem. Behav. 17, 831–836. 10.1016/0091-3057(82)90367-7 7178191

[B231] Valle-Prieto A. Conget P. A. (2010). Human mesenchymal stem cells efficiently manage oxidative stress. Stem Cells Dev. 19, 1885–1893. 10.1089/scd.2010.0093 20380515

[B232] Vasiliou V. Ziegler T. L. Bludeau P. Petersen D. R. Gonzalez F. J. Deitrich R. A. (2006). CYP2E1 and catalase influence ethanol sensitivity in the central nervous system. Pharmacogenet. Genomics 16, 51–58. 10.1097/01.fpc.0000182777.95555.56 16344722

[B233] Vetulani J. Nalepa I. Antkiewicz-Michaluk L. Sansone M. (2001). Opposite effect of simple tetrahydroisoquinolines on amphetamine- and morphine-stimulated locomotor activity in mice. J. Neural. Trans. 108, 513–526. 10.1007/s007020170053 11459073

[B234] Vinci S. Ibba F. Longoni R. Spina L. Spiga S. Acquas E. (2010). Acetaldehyde elicits ERK phosphorylation in the rat nucleus accumbens and extended amygdala. Synapse 64, 916–927. 10.1002/syn.20811 20506324

[B235] Vukojević V. Ming Y. D’Addario C. Rigler R. Johansson B. Terenius L. (2008). Ethanol/naltrexone interactions at the mu-opioid receptor. CLSM/FCS study in live cells. PLoS One 3:e4008. 10.1371/journal.pone.0004008 19104662 PMC2602977

[B236] Ward R. J. Colantuoni C. Dahchour A. Quertemont E. De Witte P. (1997). Acetaldehyde-induced changes in monoamine and amino acid extracellular microdialysate content of the nucleus accumbens. Neuropharmacology 36, 225–232. 10.1016/s0028-3908(97)00007-5 9144660

[B237] Watts P. M. Riedl A. G. Douek D. C. Edwards R. J. Boobis A. R. Jenner P.. (1998). Co-localization of P450 enzymes in the rat substantia nigra with tyrosine hydroxylase. Neuroscience 86, 511–519. 10.1016/s0306-4522(97)00649-0 9881865

[B238] Weiner H. Wang X. (1994). Aldehyde dehydrogenase and acetaldehyde metabolism. Alcohol Alcohol. 2, 141–145. 8974328

[B239] Weiner J. L. Valenzuela C. F. (2006). Ethanol modulation of GABAergic transmission: the view from the slice. Pharmacol. Ther. 111, 533–554. 10.1016/j.pharmthera.2005.11.002 16427127

[B240] Westcott J. Y. Weiner H. Shultz J. Myers R. D. (1980). *In vivo* acetaldehyde in the brain of the rat treated with ethanol. Biochem. Pharmacol. 29, 411–417. 10.1016/0006-2952(80)90521-3 7362655

[B241] Xiao C. Ye J. H. (2008). Ethanol dually modulates GABAergic synaptic transmission onto dopaminergic neurons in ventral tegmental area: role of mu-opioid receptors. Neuroscience 153, 240–248. 10.1016/j.neuroscience.2008.01.040 18343590 PMC2424267

[B242] Xiao C. Zhang J. Krnjević K. Ye J. H. (2007). Effects of ethanol on midbrain neurons: role of opioid receptors. Alcohol. Clin. Exp. Res. 31, 1106–1113. 10.1111/j.1530-0277.2007.00405.x 17577392

[B243] Xie G. Hipólito L. Zuo W. Polache A. Granero L. Krnjevic K. (2012). Salsolinol stimulates dopamine neurons in slices of posterior ventral tegmental area indirectly by activating μ-opioid receptors. J. Pharmacol. Exp. Ther. 341, 43–50. 10.1124/jpet.111.186833 22209890 PMC3310697

[B244] Xie G. Ye J.-H. (2012). Salsolinol facilitates glutamatergic transmission to dopamine neurons in the posterior ventral tegmental area of rats. PLoS One 7:e36716. 10.1371/journal.pone.0036716 22590592 PMC3349709

[B245] Zhong Y. Dong G. Luo H. Cao J. Wang C. Wu J.. (2012). Induction of brain CYP2E1 by chronic ethanol treatment and related oxidative stress in hippocampus, cerebellum and brainstem. Toxicology 302, 275–284. 10.1016/j.tox.2012.08.009 22960445

[B246] Zimatkin S. M. (1991). Histochemical study of aldehyde dehydrogenase in the rat CNS. J. Neurochem. 56, 1–11. 10.1111/j.1471-4159.1991.tb02555.x 1987314

[B247] Zimatkin S. M. Buben A. L. (2007). Ethanol oxidation in the living brain. Alcohol Alcohol. 42, 529–532. 10.1093/alcalc/agm059 17660523

[B248] Zimatkin S. M. Lindros K. O. (1996). Distribution of catalase in rat brain: aminergic neurons as possible targets for ethanol effects. Alcohol Alcohol. 31, 167–174. 10.1093/oxfordjournals.alcalc.a008128 8737012

[B249] Zimatkin S. M. Pronko S. P. Vasiliou V. Gonzalez F. J. Deitrich R. A. (2006). Enzymatic mechanisms of ethanol oxidation in the brain. Alcohol. Clin. Exp. Res. 30, 1500–1505. 10.1111/j.1530-0277.2006.00181.x 16930212

[B250] Zimatkin S. M. Rout U. K. Koivusalo M. Bühler R. Lindros K. O. (1992). Regional distribution of low-Km mitochondrial aldehyde dehydrogenase in the rat central nervous system. Alcohol. Clin. Exp. Res. 16, 1162–1167. 10.1111/j.1530-0277.1992.tb00713.x 1471772

